# Synthesis, Isolation, and Reactivity of a Phosphinidene Telluride

**DOI:** 10.1002/anie.202516494

**Published:** 2025-09-12

**Authors:** Chenyang Hu, Álvaro García‐Romero, Debanik Ray, Maren Pink, Jose M. Goicoechea

**Affiliations:** ^1^ Department of Chemistry Indiana University 800 East Kirkwood Ave. Bloomington Indiana 47405 USA; ^2^ Department of Chemistry Chemistry Research Laboratory University of Oxford 12 Mansfield Rd. Oxford OX1 3TA UK

**Keywords:** Metathesis, Multiple bonds, Phosphinidenes, Phosphorus, Tellurium

## Abstract

The synthesis and isolation of a phosphorus(III) telluride is reported. The compound was accessed by means of a double bond metathesis reaction between an amido‐functionalized phosphinidene oxide, BnArNP═O (Ar = bulky aryl substituent), with a silylene telluride, L(NMe_2_)Si═Te (L = PhC(N*^t^* Bu)_2_). The resulting phosphinidene telluride, BnArNP═Te, exhibits a short P═Te bond (2.297(1) Å), resulting from a significant p_π_–p_π_ orbital interaction between the phosphorus(III) and tellurium(−II) atoms. Key to the stabilization of this compound is the amido substituent associated with the phosphorus atom, which provides kinetic stabilization due to its steric bulk but also significant thermodynamic stabilization through negative hyperconjugation of the nitrogen atom lone pair into antibonding orbital interactions between phosphorus and tellurium. The title compound acts as a versatile phosphinidene transfer platform, accompanied by the extrusion of elemental tellurium to afford other phosphorus(III) compounds via transient phosphorus(V) intermediates.

## Graphical abstract

We describe the synthesis and isolation of a phosphinidene telluride. This species acts as a versatile phosphinidene transfer reagent accompanied by the extrusion of elemental tellurium.

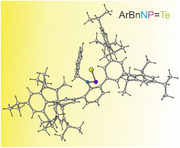

## Introduction

**Figure 1:**
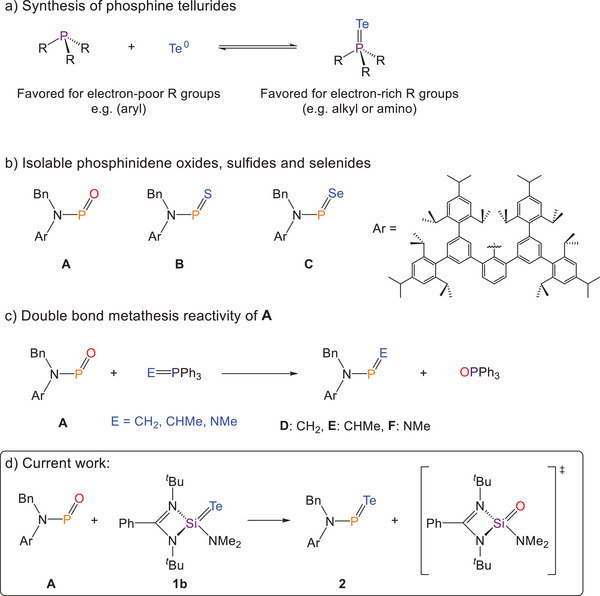
a) Synthesis of phosphorus(V) tellurides; b) phosphinidene oxides, sulfides, and selenides; c) reactivity of **A**; and d) present work; synthesis of a phosphinidene telluride.

The first phosphorus(V) telluride—the phosphine telluride *^n^* Bu_3_P═Te—was reported in 1963 by the reaction of tri‐*n*‐butylphosphine with elemental tellurium.^[^ ^1^ ^]^ In the ensuing six decades, a limited number of such compounds have been described, primarily derived from electron‐rich phosphines (bearing alkyl‐ or amino‐substituents).^[^ ^2^, ^3^, ^4^, ^5^, ^6^, ^7^, ^8^, ^9^, ^10^, ^11^, ^12^, ^13^, ^14^, ^15^, ^16^, ^17^, ^18^ ^]^ The general instability of phosphine tellurides is attributed to the redox mismatch between the phosphorus(V) and tellurium(−II) oxidation states. When electron‐deficient phosphines such as triphenylphosphine (PPh_3_) are reacted with tellurium, the resulting tellurides tend to undergo reversible dissociation to the free phosphine and elemental tellurium (Figure 1a).^[^ ^19^, ^20^ ^]^ This dynamic exchange is observable by multielement NMR spectroscopy; for instance, upon the addition of excess phosphine (PR_3_) to a solution of R_3_P═Te, the characteristic doublet observed in the ^125^Te NMR spectrum collapses, indicating a rapid exchange process.^[^ ^21^, ^22^ ^]^ Du Mont and Kroth proposed that the exchange proceeds via linear intermediates of the type R_3_P═Te─PR_3_,^[^ ^23^ ^]^ the likes of which have been structurally authenticated for Ph_3_P═Te─PPh_3_.^[^ ^19^ ^]^ A detailed variable‐temperature and concentration‐dependent NMR study of the acyclic P(III)/P(V) monotelluride *^i^* Pr_2_PCH_2_P(Te)*^i^* Pr_2_ revealed that the intermolecular exchange has an energy barrier of 21.9(32) kJ mol^−1^.^[^ ^13^ ^]^

The inherent lability of the P(V)═Te bond has enabled the use of phosphine tellurides as precursors for the synthesis of materials, including colloidal quantum dots.^[^ ^24^, ^25^, ^26^ ^]^ Specifically, mixtures of elemental tellurium and trioctylphosphine (TOP) have been widely applied in the preparation of colloids of various metal tellurides, including those of cadmium,^[^ ^27^, ^28^ ^]^ lead,^[^ ^29^ ^]^ mercury,^[^ ^30^, ^31^ ^]^ tin,^[^ ^32^ ^]^ silver,^[^ ^33^ ^]^ or copper.^[^ ^34^ ^]^ In these processes, phosphine tellurides function as tellurium transfer reagents. The phosphine ligands facilitate the solubilization of elemental tellurium and enable its redeposition as Te^2−^ at elevated temperatures, resulting in the formation of nanocrystals with well‐defined size, morphology, and crystal structure. Nonetheless, due to the elusive nature of phosphine tellurides, their chemical behavior remains poorly understood, which in turn leads to limited reproducibility and challenges in process control.^[^ ^35^, ^36^ ^]^ This has prompted a continued interest in developing isolable, well‐characterized phosphine telluride complexes to provide greater predictability and precision in quantum dot synthesis.^[^ ^25^ ^]^

Phosphorus(III) tellurides—phosphinidene tellurides, RP═Te—are fundamentally interesting compounds that in principle could also be used as a soluble, stoichiometric tellurium sources. However, while lighter phosphinidene chalcogenides (O, S, Se) have been studied extensively in inert gas matrices,^[^ ^37^, ^38^, ^39^ ^]^ or isolated using trapping experiments,^[^ ^40^, ^41^, ^42^, ^43^, ^44^, ^45^, ^46^, ^47^, ^48^, ^49^, ^50^ ^]^ they are extremely difficult to isolate. Phosphinidene sulfides and selenides were first reported by Schmidpeter and co‐workers in the early 1990s,^[^ ^51^, ^52^ ^]^ however their tellurium‐containing analogues were, until very recently, entirely inaccessible.^[^ ^53^, ^54^ ^]^ The dearth of phosphinidene tellurides in the literature is largely attributed to the lack of appropriate precursors capable of cleanly generating such species. Lighter phosphinidene chalcogenides are typically generated in situ through thermolysis/photolysis of phosphorus(V) chalcogenides with labile leaving groups (e.g., Stille's 3‐benzo‐1,4,5,6,7‐pentaphenyl‐7‐phosphabicyclo[2.2.1]hept‐5‐ene oxide)^[^ ^55^ ^]^ or reductive treatment of phosphonic or phosphonothioic dichlorides (e.g., RCl_2_P═E, where E = O, S); however, due to the limited access to isolable phosphine tellurides, there are no such precursors for the synthesis of phosphinidene tellurides.

Recently, we reported the isolation of a phosphinidene oxide supported by an extremely bulky amino group (**A**; Figure 1b) and its transformation to a phosphinidene sulfide (**B**) and selenide (**C**).^[^ ^56^, ^57^ ^]^ Phosphinidene oxide **A** undergoes phospha‐(aza)‐Wittig reactivity with ylides, offering a new pathway to a range of phosphorus‐main group element multiple bonds via double bond metathesis (Figure 1c).^[^ ^58^ ^]^ Alkene metathesis is widely used in organic synthesis, in both academic and industrial contexts;^[^ ^59^, ^60^, ^61^, ^62^ ^]^ however, such reactivity is rarely observed for multiple bonds between the heavier main‐group elements. This scarcity is largely due to the relative dearth of heavier alkene analogues. In such systems, the formation of two σ bonds is typically energetically favored over a σ + π bond. As a result, multiple bonds between the heavier main‐group elements often undergo cycloaddition‐type reactivity rather than metathesis. Nevertheless, several examples of multiple bond metathesis for compounds of the main‐group elements have been reported to date,^[^ ^63^ ^]^ including some elegant studies on Ge═Ge bond metathesis reported by Scheschkewitz.^[^ ^64^ ^]^ These findings prompted us to explore the synthesis of a phosphinidene telluride using a double‐bond metathesis strategy. Herein, we describe the double bond metathesis reaction between phosphinidene oxide **A** and a silylene telluride **1b**, generating the corresponding phosphinidene telluride **2** and silylene oxide.

## Results and Discussion

### Synthesis of a Phosphinidene Telluride

As mentioned above, the phosphinidene oxide **A** readily undergoes metathesis reactions with ylides; however, we also observed that it is unreactive toward phosphine sulfides S═PR_3_ (R = Ph or Me). This suggests that the polarity of the substrate E═P bond (E = CH_2_, CHMe, NMe, S) plays an important role in dictating reaction spontaneity. This observation led us to hypothesize that the use of a reagent with a more polarized Ch═E bond (Ch = chalcogen; E = main‐group element) could facilitate the desired metathesis reaction between the P═O and the E═Ch bond, and that such a strategy could be employed for the synthesis of an elusive phosphinidene telluride (RP═Te). Recent developments in the chemistry of silylenes (:Si^II^R_2_) have shown that they are strong σ‐donors, often surpassing phosphines as nucleophiles.^[^ ^65^ ^]^ Their oxidized counterparts, silylene chalcogenides (Ch═Si^IV^R_2_), are expected to possess greater polarity compared to phosphine chalcogenides, owing to the more electropositive character of silicon (1.90) relative to phosphorus (2.19).^[^ ^66^ ^]^ We reasoned that such species could be used as metathesis partners with **A** to access phosphinidene tellurides. Among available candidates, we identified amidinate‐ligated silylene tellurides as a viable precursor. These species are synthetically well‐established,^[^ ^67^ ^]^ and the silicon center in these compounds is more sterically accessible than that of other reported silylenes. This structural feature allows the Si═Te double bond to be accommodated within the binding pocket of our *meta*‐substituted terphenyl ligand, providing a suitable framework for engaging in metathesis with the P═O bond of phosphinidene oxide **A**.

**Scheme 1:**
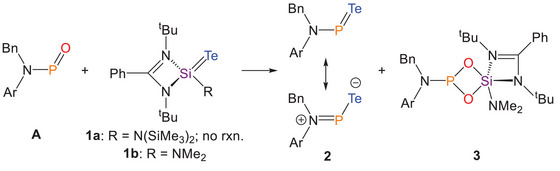
Synthesis of compounds **2** and **3**.

Our investigation commenced with the reported bis(trimethylsilyl)amide‐substituted silylene telluride **1a**. However, due to the significant steric bulk of the bis(trimethylsilyl)amide moiety, no reaction was observed between **1a** and phosphinidene oxide **A**, even upon heating. To reduce steric hindrance, a less bulky analogue, **1b**, bearing a dimethylamide substituent, was synthesized. Gratifyingly, treatment of **A** with an excess of **1b** (Scheme 1) led to the immediate consumption of **A** and formation of three new species, as evidenced by in situ ^31^P{^1^H} NMR spectroscopy. Two broad resonances at 633.9 and 625.0 ppm were observed in the ^31^P{^1^H} NMR spectrum, consistent with the formation of phosphinidene telluride **2**, analogous to those previously reported for the phosphinidene chalcogenides **A**, **B**, and **C**. The presence of two distinct resonances is attributed to *cis*–*trans* isomerism arising from restricted rotation about the N─P bond. Another sharp singlet was also observed in ^31^P{^1^H} NMR spectrum at 133.1 ppm, which integrates in a 1:1 ratio relative to the resonances attributed to **2**. Drawing on precedent from the synthesis of **B** and **C**, we postulate that this resonance corresponds to a chalcogenide‐bridged dimeric species, incorporating both the phosphinidene and silylene moieties. Indeed, this species, designated as compound **3**, features a doublet at −100.6 ppm with a coupling constant of ^3^ *J*_P─Si_ = 8.4 Hz in its ^29^Si{^1^H} NMR spectrum. It is worth noting that such a ^29^Si{^1^H} chemical shift is similar to those reported for [LSi(X)(μ‐O)]_2_ (L = PhC(N*^t^* Bu)_2_; X = PPh_2_, NPh_2_, NMe_2_, O*^t^* Bu).^[^ ^68^ ^]^

**Figure 2:**
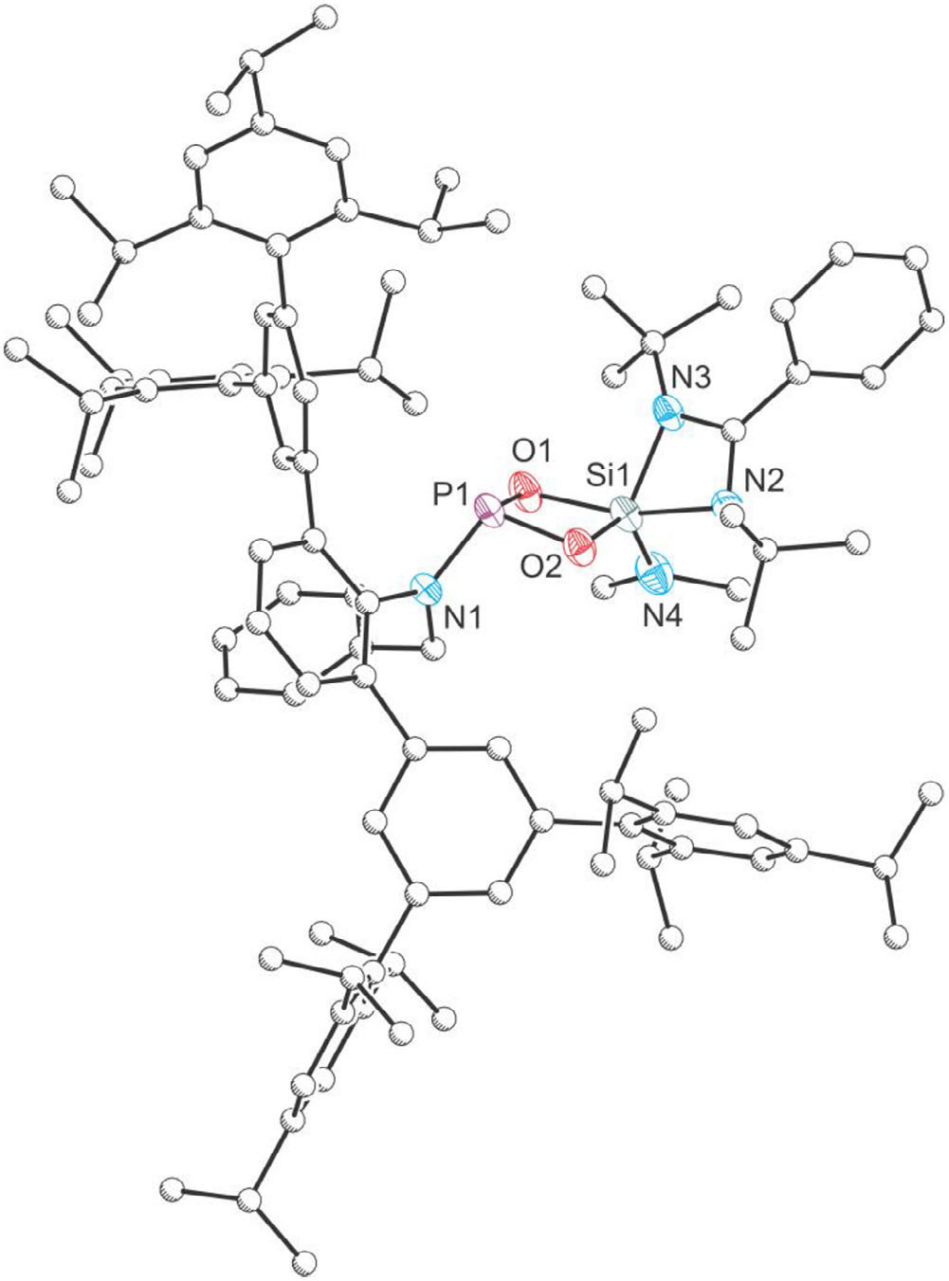
Single‐crystal X‐ray structure of **3**. Carbon atoms depicted with an arbitrary radius. Thermal ellipsoids are pictured at a 50% probability level. Hydrogen atoms are removed for clarity.

The structure of compound **3** was confirmed by single‐crystal X‐ray diffraction (Figure 2).^[^ ^69^ ^]^ The structure reveals a four‐membered P─O─Si─O ring featuring a three‐coordinate phosphorus center and a five‐coordinate silicon atom (τ_5_ = 0.55).^[^ ^70^ ^]^ The P1─O1 and P1─O2 bond lengths were determined to be 1.632(2) and 1.653(2) Å, respectively, both of which are slightly shorter than the value expected for a P─O single bond (1.74 Å).^[^ ^71^ ^]^ This bond shortening is likely attributed to ring strain within the four‐membered ring, a structural feature previously reported for dimeric phosphinidene oxides.^[^ ^72^ ^]^ The five‐coordinate silicon center closely resembles the geometry of reported [LSi(X)(μ‐O)]_2_ compounds (L = PhC(N*^t^* Bu)_2_; X = PPh_2_, NPh_2_, NMe_2_, O*^t^* Bu),^[^ ^68^ ^]^ with Si─O bond lengths of 1.764(2) and 1.709(2) Å.

**Figure 3:**
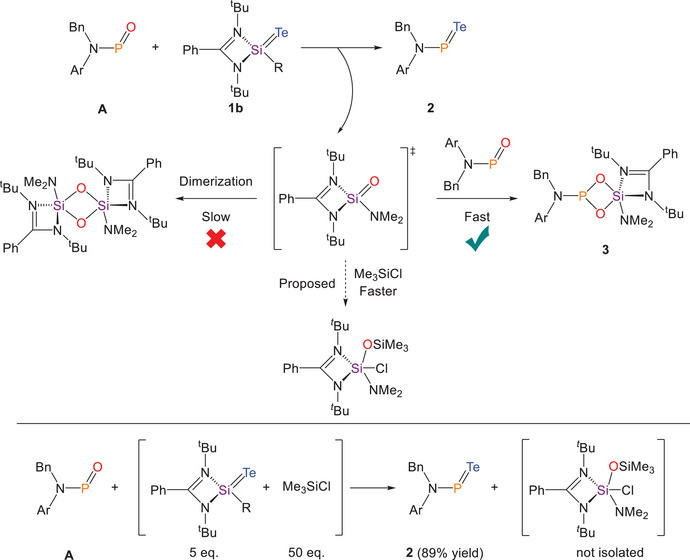
Top: Proposed reaction pathway for the metathesis reaction between **A** and **1b**. Bottom: Optimized synthesis of **2**.

Confirmation of the structure of **3**, formed during the metathesis reaction between **A** and **1b**, provided important mechanistic insight into the formation of compound **2**. After the first metathesis step, one equivalent of LSi(O)(NMe_2_) is generated as a by‐product. Instead of undergoing self‐dimerization to form [LSi(NMe_2_)(μ‐O)]_2_, as previously reported,^[^ ^68^ ^]^ the silicon oxide LSi(O)(NMe_2_) reacts with a second equivalent of the phosphinidene oxide **A**, resulting in the formation of compound **3** (Figure 3, top). This distinct selectivity presumably rises from significant differences in reaction rates, as the dimerization is irreversible. Although metathesis cleanly affords the desired phosphinidene telluride **2**, the formation of side‐product **3** limits the theoretical yield of **2**, since half of **A** is diverted to the side reaction. Moreover, due to the similar solubility profiles of compounds **2** and **3**, the resulting mixture complicates the purification and isolation of the targeted product. To circumvent this issue, we reasoned that LSi(O)(NMe_2_) must be removed from the reaction mixture prior to its interception by **A**. In order to achieve this, the addition of excess trimethylsilyl chloride Me_3_SiCl was proposed to trap the in situ generated product LSi(O)(NMe_2_), thereby suppressing the undesired side reaction and improving the overall efficiency of phosphinidene telluride formation.

To evaluate our hypothesis, 5 equivalents of **1b** and 50 equivalents of trimethylsilyl chloride Me_3_SiCl were pre‐mixed in benzene before a benzene solution of **A** was added to it dropwise (Figure 3, bottom). In situ ^31^P{^1^H} NMR spectroscopy revealed clean conversion of **A** into the desired phosphinidene telluride **2**, with no detectable formation of side‐product **3**. This modified protocol was successfully scaled to an 80 mg batch, delivering compound **2** in excellent yield (89%). Following isolation, an extensive multinuclear NMR spectroscopic analysis was performed on compound **2**. As mentioned, the ^31^P{^1^H} NMR spectrum of **2** revealed two broad peaks at 633.9 and 625.0 ppm; we attributed the broadening of these peaks to the chemical exchange between *cis*‐ and *trans*‐isomers, as observed previously for the aminoiminophosphane **F**, which features the same ligand backbone.^[^ ^58^ ^]^ However, variable‐temperature ^31^P{^1^H} NMR experiments conducted between 25 and 70 °C showed no significant change in line‐width, suggesting that interconversion between isomers is slow on the ^31^P{^1^H} NMR timescale in this temperature range. Interestingly, no signal was observed in the ^125^Te NMR spectrum, whether at ambient temperature, low temperature (−45 °C), or elevated temperature (70 °C). The absence of a ^125^Te NMR resonance may be attributed to chemical exchange between the *cis*‐ and *trans*‐ isomers of **2**. Nevertheless, ^125^Te satellites were observed in prolonged ^31^P{^1^H} NMR acquisitions, revealing a ^1^ *J*_P─Te_ coupling constant of 1747.8 Hz, consistent with values reported for phosphine tellurides.^[^ ^20^ ^]^ Notably, these satellites were consistently observed across the entire temperature range studied (25 to 70 °C), ruling out rapid dissociation into free phosphinidene and elemental tellurium at elevated temperature. The NMR data obtained for **2** compare favorably with the values recently reported by Tan for their phosphinidene telluride (^31^P NMR: 892.4 ppm; ^1^ *J*_P─Te_ of 2045.7 Hz).^[^ ^54^ ^]^

**Figure 4:**
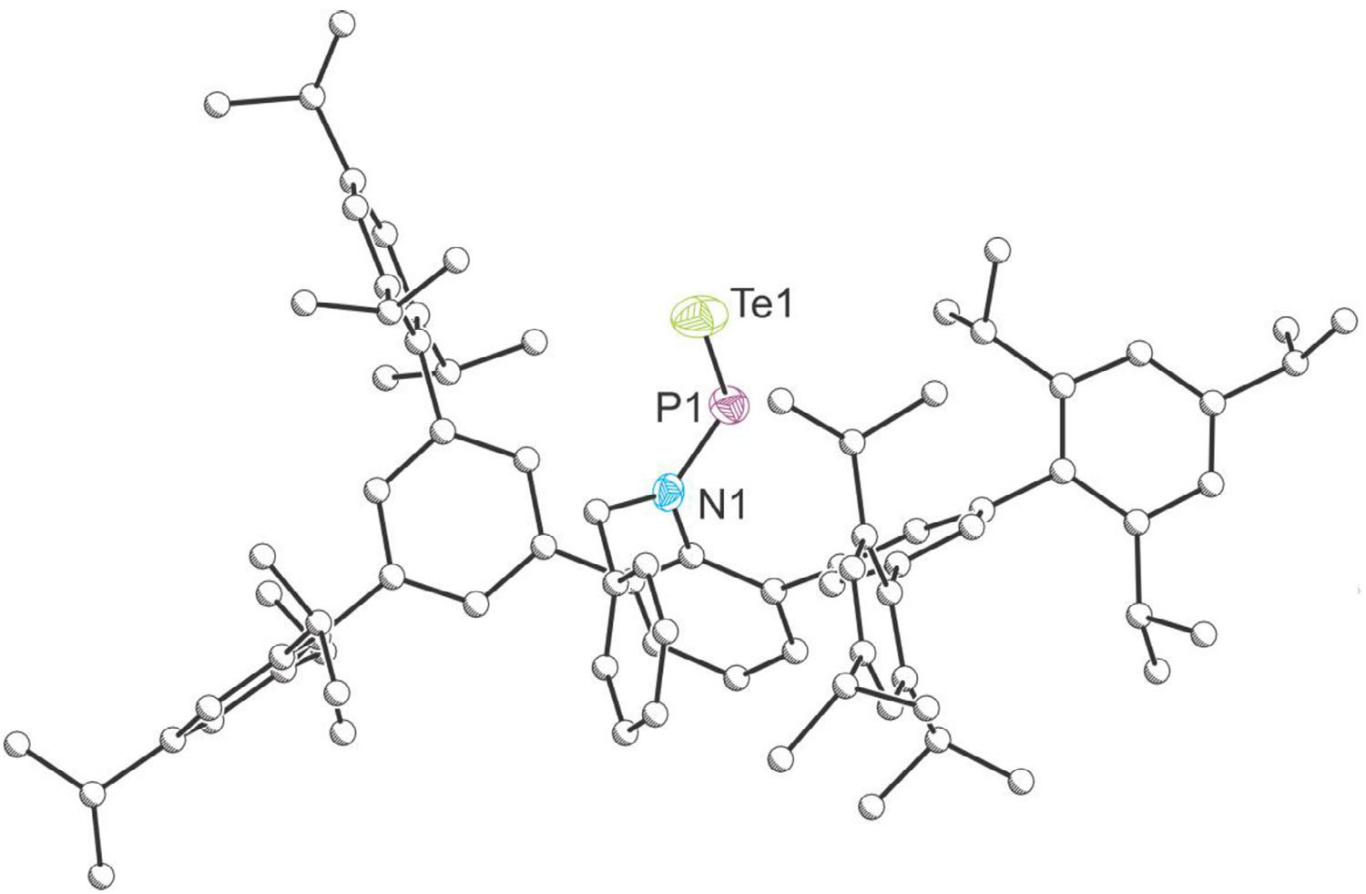
Single‐crystal X‐ray structure of **2**. Carbon atoms depicted with an arbitrary radius. Thermal ellipsoids are pictured at a 50% probability level. Hydrogen atoms are removed for clarity.

Structural confirmation of **2** was obtained through single‐crystal X‐ray diffraction analysis (Figure 4). The crystal structure revealed a *trans*‐geometry about the N─P bond, closely resembling the geometry exhibited by **A–C**. The measured P═Te bond length of 2.297(1) Å aligns well with the computationally predicted value of 2.30 Å for a P═Te double bond.^[^ ^73^ ^]^ This value is comparable to that recently reported by Tan for his phosphinidene telluride (2.2853(18) Å).^[^ ^54^ ^]^ Similar to the corresponding sulfide and selenide congeners (**B** and **C**), the P═Te bond in **2** is noticeably shorter than those typically found in phosphine tellurides (R_3_P═Te), which generally range from 2.34 to 2.38 Å.^[^ ^20^ ^]^ This bond contraction is attributed to a significant p_π_–p_π_ interaction between the phosphorus and tellurium atoms. In contrast, conventional phosphine tellurides are characterized primarily by p→σ* hyperconjugative interactions, wherein the tellurium lone pairs donate into two degenerate σ*‐orbitals of the P─R bonds. The planar geometry of the nitrogen atom (Σ°_N_: 360.0°) and short N─P bond length (1.669(2) Å) indicate the donation from the nitrogen lone pair to the P─Te π* orbital. This delocalization is in line with the observed ^31^P{^1^H} NMR spectrum, as both *cis*‐ and *trans*‐isomers are observed, indicating restricted rotation about the N─P bond.

**Figure 5:**
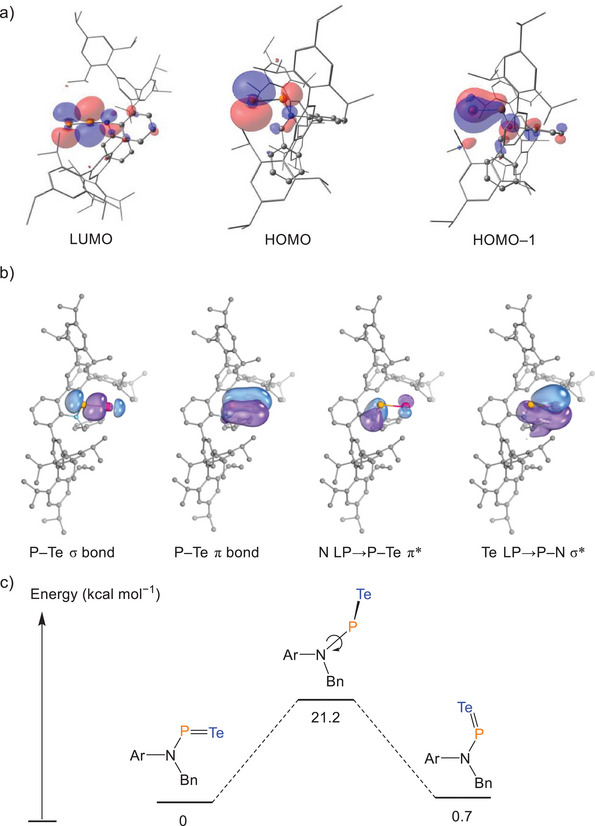
a) Selected Kohn–Sham molecular orbital depictions (isovalue = 0.03) for compound **2**; b) depiction of selected IBOs of **2**, orbital isosurfaces enclose 80% of the integrated electron density of the orbital; c) calculated N─P bond rotation energy profile for **2**.

To further elucidate the electronic structure of **2**, density functional theory (DFT) calculations were performed. The geometry optimized at the M06‐2X/Def2‐SVP level showed excellent agreement with the crystallographic data (e.g., P─Te: 2.312 Å; N─P: 1.685 Å; Σ°_N_: 359.8°). Kohn–Sham molecular orbital analysis clearly supports the presence of P═Te double bond, with HOMO–1 corresponding to a localized P─Te π‐bonding orbital and the LUMO representing the P─Te π* antibonding orbital (Figure 5a). The HOMO has tellurium lone pair character. This bonding picture is further corroborated by intrinsic bond orbital (IBO) analysis, which identifies distinct σ‐ and π‐type bonding interactions between the phosphorus and tellurium atoms (Figure 5b). The tellurium atom bears two extra lone pairs, one of which is polarized toward N─P bond (Te contribution 67.3%, P contribution 14.5%, and N contribution 6.7%), indicating negative hyperconjugation from the lone pair of tellurium to N─P σ* orbital. Similarly, the lone pair of the nitrogen atom is found to be polarized toward P─Te bonds (N contribution 78.0%, Te contribution 13.5%, and P contribution 4.1%), representing negative hyperconjugation into the P─Te π* orbital; such interactions have also been found in its lighter congeners **A**, **B**, and **C**. Such negative hyperconjugation interactions are known to play an important role in the stabilization of electronically interesting compounds such as a σ^0^π^2^ carbene.^[^ ^74^, ^75^ ^]^ Computed Mayer bond indices revealed a bond order of 1.72 for P═Te bond and 1.13 for P─N bond, which is in line with the proposed P═Te double bond and delocalization among N─P─Te moiety. The calculated rotational energy barrier for N─P bond rotation at the SMD‐M06‐2X/def2‐TZVP//M06‐2X/def2‐SVP level is 21.2 kcal mol^−1^, closely matching the values computed for the lighter chalcogen analogues. This relatively high rotational barrier is attributed to both the multiple bonding character of N─P bond and the longer P═Te bond compared to the P═Ch bond in **A**, **B**, and **C**, which leads to extra steric hindrance during rotation. The *trans*‐isomer of **2** is thermodynamically favored by 0.7 kcal mol^−1^, in agreement with the observed crystal structure (Figure 5c).

Owing to the intense green color of solutions of compound **2**, a UV–vis spectroscopic analysis was conducted. The spectrum revealed a strong absorption band at λ_max_ = 435 nm (see Supporting Information). Time‐dependent DFT (TD‐DFT) calculations reproduced the experimental data reasonably well, predicting an absorption at λ_calc_ = 394 nm. The calculations indicated that this transition originates from the HOMO–1 to LUMO excitation, which corresponds to a π → π* transition primarily involving the P═Te bond.

### Reactivity Studies

**Scheme 2:**
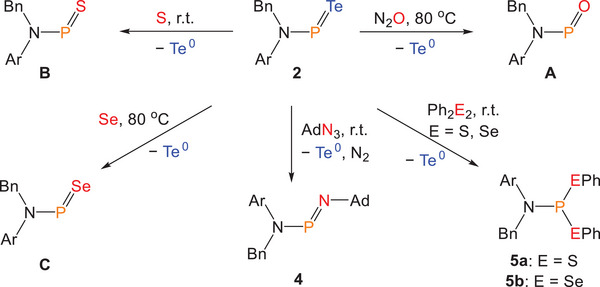
Reactivity of **2** toward oxidizing agents.

**Figure 6:**
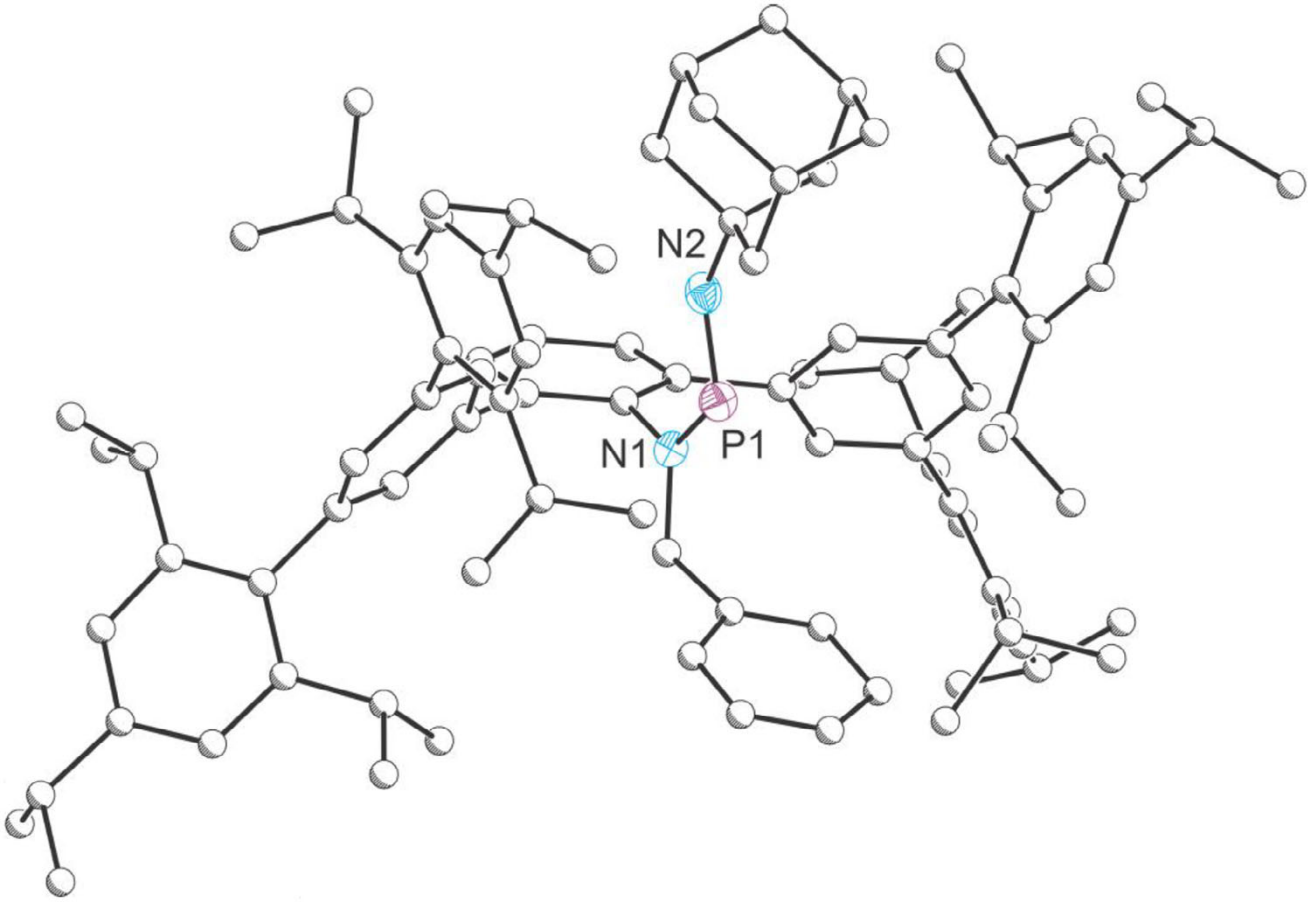
Single‐crystal X‐ray structure of **4**. Carbon atoms depicted with an arbitrary radius. Thermal ellipsoids are pictured at a 50% probability level. Minor positionally disordered components and hydrogen atoms are removed for clarity.

Further reactivity studies revealed that compound **2** can serve as an effective phosphinidene transfer reagent. Upon treatment with oxidants such as N_2_O, sulfur, or selenium, either at room temperature or upon mild heating, immediate formation of elemental tellurium was observed, evidenced by the appearance of a black precipitate in the reaction mixture. In situ ^31^P{^1^H} NMR spectroscopy studies confirmed the formation of corresponding phosphinidene chalcogenides **A**, **B**, and **C**, all of which can be isolated in good yields after work‐up (Scheme 2; **A**: 73%; **B**: 80%; **C**: 71%). Additionally, organic azides were found to function as nitrene transfer reagents, enabling substitution of the telluride moiety in **2**. When benzene was added to a solid mixture of adamantyl azide (AdN_3_) and compound **2**, a black precipitate formed once again, consistent with the elimination of elemental tellurium. In situ ^31^P{^1^H} NMR spectroscopy revealed a broad resonance at 316.4 ppm assigned to the newly formed compound **4**, which closely resembles previously reported aminoiminophosphanes **F** formed via a phospha‐aza‐Wittig reaction.^[^ ^58^ ^]^ The structure of **4** was confirmed through single‐crystal X‐ray diffraction (Figure 6). The crystal structure of **4** revealed a *cis*‐geometry about the N─P bond, which contrasts with the *trans*‐geometries observed for **A**, **B**, **C**, and **F**. We attribute this difference to the bulky adamantyl group, as the *cis*‐geometry in **4** minimizes the steric hindrance. All other bond metrics are comparable to those previously reported for **F**.

**Scheme 3:**
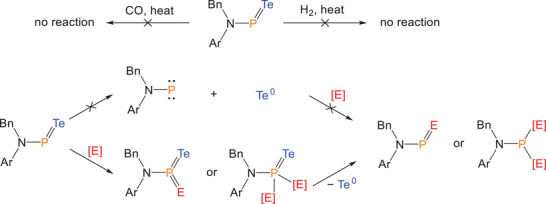
Proposed reaction pathways for **2**.

While two‐coordinate phosphorus(III) compounds can be readily accessed from **2**, the synthesis of three‐coordinate species is also feasible via oxidative addition to the phosphorus center, analogous to the reactivity observed for [K(18‐crown‐6)][ArNPO].^[^ ^56^ ^]^ Treatment of **1** with diphenyl disulfide S_2_Ph_2_ or diphenyl diselenide Se_2_Ph_2_ led to the instantaneous formation of elemental tellurium (Scheme 3), as indicated by the appearance of a black precipitate. In situ ^31^P{^1^H} NMR spectroscopy revealed a broad resonance at 167.1 ppm for **5a** and 191.5 ppm for **5b**. To probe the origin of the line broadening in the ^31^P{^1^H} NMR spectrum, variable‐temperature NMR experiments were conducted on compound **5a** (see Supporting Information for full details). Upon heating, the broad phosphorus resonance sharpened into a single well‐defined resonance, while cooling led to splitting into two broad peaks. A similar temperature‐dependent behavior was observed in the ^1^H NMR spectrum, particularly for two sets of resonances assigned to the *^i^* Pr groups and aromatic protons. Based on these variable temperature NMR spectroscopy studies, we can draw the conclusion that within the bulky aromatic ligand pocket, the phenyl group bonded to sulfur has close contact with an inner *^i^* Pr group, which results in restricted rotation of the phenyl group and a concomitant broadening of the resonances in the ^31^P{^1^H} and ^1^H NMR spectra. A similar effect was also observed in variable temperature NMR spectra of **5b**. Despite numerous attempts to obtain single‐crystal diffraction data for **5a** and **5b**, no satisfactory crystals could be grown for structural confirmation.

While oxidizing reagents readily react with compound **2** to mediate phosphinidene transfer reactions, reducing reagents such as carbon monoxide CO and hydrogen (H_2_) give rise to no reaction, even under elevated temperatures (Scheme 3). To rationalize the mechanism of phosphinidene transfer from compound **2**, two plausible pathways were considered. The first mechanism involves homolytic cleavage of the P═Te double bond, generating a free phosphinidene intermediate, which can subsequently undergo oxidation by external reagents. The second scenario entails direct oxidation of **2** to form a transient phosphorus(V) telluride species, which, due to its inherent instability (as discussed previously), would readily eliminate elemental tellurium Te^0^, affording the final phosphinidene transfer product. Given the complete inertness of compound **2** toward CO and H_2_, the second mechanism appears more likely. If a free phosphinidene intermediate were indeed formed during the reaction, it would be expected to undergo facile reaction with CO or H_2_, yielding known products such as phosphaketenes (R─P═C═O) or primary phosphines (R─PH_2_), based on previously reported masked‐phosphinidene reactivity.^[^ ^76^, ^77^ ^]^

## Conclusion

Following the successful isolation of a phosphinidene oxide **A**, sulfide **B**, and selenide **C**, we herein report a missing piece in this phosphinidene chalcogenide family, the phosphinidene telluride **2**. This species was obtained via a double bond metathesis reaction between phosphinidene oxide **A** and silylene telluride **1b**. Owing to the high reactivity of the silylene oxide, it can either undergo side reactions with **A** or be selectively trapped by trimethylsilyl chloride Me_3_SiCl. Utilizing this trapping strategy, a one‐pot synthesis of phosphinidene telluride **2** was achieved on an 80 mg scale, with Me_3_SiCl serving as an effective additive to suppress undesired pathways. Single‐crystal X‐ray diffraction analysis revealed that **2** adopts a *trans*‐geometry across the R_2_N─P moiety, closely resembling the solid‐state structures of its lighter congeners **A–C**. DFT calculations showed that the nitrogen lone pair engages in donation to antibonding P─Te orbitals. This interaction contributes to a restricted rotation about the N─P bond, with a computed rotational barrier of 21.2 kcal mol^−1^. Further reactivity studies demonstrated that **2** functions as a phosphinidene transfer reagent under oxidative conditions. The mechanistic pathway likely proceeds via a phosphorus(V) telluride intermediate, which, due to its instability, rapidly eliminates elemental tellurium Te^0^ to furnish the phosphinidene transfer product. The labile nature of the telluride moiety in **2** underscores the potential utility of phosphinidene tellurides in tellurium‐containing materials synthesis and offers a platform for exploring new reactivity modes at the P═Te bond.

## Supporting Information

The authors have cited additional references within the Supporting Information.^[^ ^78^, ^79^, ^80^, ^81^, ^82^, ^83^, ^84^, ^85^, ^86^, ^87^, ^88^, ^89^, ^90^, ^91^, ^92^, ^93^, ^94^, ^95^, ^96^, ^97^, ^98^, ^99^, ^100^, ^101^, ^102^ ^]^

## Conflict of Interests

The authors declare no conflict of interest.

## Supporting information



Supporting Information

## Data Availability

The data that support the findings of this study are available in the Supporting Information of this article.
