# Supplementary material for: Synthesis, Isolation, and Reactivity of a Phosphinidene Telluride
Source: Angew Chem Int Ed Engl. 2025 Sep 12;64(45):e202516494. doi: 10.1002/anie.202516494 (PMC12582002; doi:10.1002/anie.202516494)
Supplement: Supplementary file 1 — Supporting Information [file ANIE-64-e202516494-s001.docx]

*Supporting information*

**Synthesis, Isolation and Reactivity of a Phosphinidene Telluride**

Chenyang Hu,^†,‡^ Álvaro García-Romero, ^†^ Debanik Ray, ^†^ Maren Pink,^†^ and Jose M. Goicoechea^†,^*

^†^ Department of Chemistry, Indiana University, 800 East Kirkwood Ave., Bloomington, Indiana, 47405, U.S.A.

^‡^ Department of Chemistry, University of Oxford, Chemistry Research Laboratory, 12 Mansfield Rd., Oxford, OX1 3TA, U.K.

E-mail: [jgoicoec@iu.edu](mailto:jgoicoec@iu.edu)

**Contents**

[1. Experimental details 2](#_Toc204322183)

[2. NMR spectra 17](#_Toc204322184)

[3. High Resolution Mass Spectra 28](#_Toc204322185)

[4. Variable Temperature NMR studies of compound **2**, **4**, **5a** and **5b** 30](#_Toc204322186)

[5. UV-vis Spectrum of **2** 35](#_Toc204322187)

[6. X-ray Crystallographic Data 36](#_Toc204322188)

[7. Computational Details 48](#_Toc204322189)

[8. References 49](#_Toc204322190)

# **1. Experimental details**

**1.1. General Synthetic Methods**

All reactions and product manipulations were carried out under an inert atmosphere of argon or dinitrogen using standard Schlenk-line or glovebox techniques (MBraun UNIlab glovebox maintained at < 0.1 ppm H_2_O and < 0.1 ppm O_2_). Benzene (anhydrous, Sigma Aldrich), toluene (Fisher Chemical, HPLC grade), hexane (Fisher Chemical, HPLC grade) and acetonitrile (Fisher Chemical, HPLC grade) were purified using a Pure Process Technology (PPT) solvent purification system (SPS). C_6_D_6_ (Aldrich, 99.5%) and *d*_8_-toluene (Aldrich, 99%) were distilled over sodium/benzophenone. All dry solvents were stored under argon in gas-tight ampoules over activated 3 Å molecular sieves. Compounds **A**,^[56]^ **1a,**^[67]^ and LSi(NMe_2_) (L = PhC(N*^t^*Bu)_2_) were synthesized following procedures described in the literature.^[78]^

**Additional characterization techniques:** NMR spectra were acquired on a Bruker 500 MHz Avance Neo, a Varian 500 MHz Inova, or a Varian 400 MHz Inova NMR spectrometer. Chemical shifts (δ) are reported in parts per million (ppm). ^1^H and ^13^C NMR spectra are referenced to TMS using residual protio-solvent resonance (^1^H NMR C_6_D_6_: δ = 7.16 ppm, ^13^C NMR C_6_D_6_: δ = 128.06 ppm; ^1^H NMR *d*_8_-toluene: δ = 2.08 ppm). ^29^Si NMR spectra were externally referenced to TMS. ^31^P NMR spectra were externally referenced to an 85% solution of H_3_PO_4_ in H_2_O. ^125^Te NMR spectra were externally referenced to a solution of TeMe_2_ in C_6_D_6_. High-resolution mass spectra were recorded on a Thermo Q-Exactive Plus (ESI-TOF, positive ion mode) instrument at the Mass Spectrometry Facility of the Department of Chemistry of Indiana University.

**1.2. Synthesis of 1b**

**Scheme S1.** Synthesis of 1**b.**

To a solid mixture of LSi(NMe_2_) (L = PhC(N*^t^*Bu)_2_; 100 mg, 0.33 mmol; 1 eq.) and elemental tellurium (63.2 mg, 0.49 mmol; 1.5 eq.), benzene (2 mL) was added. The mixture was stirred overnight at room temperature before it was filtered. The filtrate was dried *in vacuo* and subsequently redissolved in a toluene-hexane (1:1) mixture. The mixture was left in a –35 °C freezer overnight to crystalize. The supernatant was removed, and the resulting solid was dried *in vacuo* to afford **1b** (83 mg, 0.019 mmol; 58% yield) as a colorless powder. Single crystals of **1b** suitable for X-ray diffraction were obtained by leaving a toluene/hexane solution of **1b** in a –35 °C freezer overnight.

**^1^H NMR (500 MHz, C_6_D_6_):** δ (ppm) 1.13 (s, 18H; NC(C*H*_3_)_3_), 2.79 (s, 6H; N(C*H*_3_)_2_), 6.70 (d, ^3^*J*_H–H_ = 7.7 Hz, 1H; Ar*H*), 6.79–6.91 (m, 4H; Ar*H*).

**^13^C{^1^H} NMR (125 MHz, C_6_D_6_):** δ (ppm) 31.07 (s; NC(*C*H_3_)_3_), 37.88 (s; N*C*(CH_3_)_3_), 54.89 (s; N(*C*H_3_)_2_), 127.33, 128.71, 130.39, 131.06 (s; Ar*C*), 173.57 (s; N*C*N).

**^29^Si{^1^H} NMR (99.4 MHz, C_6_D_6_):** δ (ppm) –23.69 (s).

**HRMS (*m*/*z*):** [M+H]^+^ Calcd. for C_17_H_30_N_3_Si_1_Te_1_, 434.1266; Found 434.1266.

**1.3. Synthesis and purification of 3**

**Scheme S2.** Synthesis and purification of **3**.

To a solid mixture of **A** (10 mg, 0.0084 mmol; 1 eq.) and **1b** (3.6 mg, 0.0084 mmol; 1 eq.), benzene (0.7 mL) was added. The mixture was stirred for 1 min at room temperature and monitored by ^31^P{^1^H} NMR spectroscopy. Once compound **A** was fully consumed, the mixture was dried *in vacuo*. The green residue was washed with MeCN (1.5 ml × 3) until the solid was colorless. The resulting solid was dried *in vacuo* to afford **3** (4.8 mg, 0.0032 mmol; 38% yield based on **A**) as a white powder. Single crystals of **3** suitable for X-ray diffraction were obtained by slow evaporation of a concentrated hexane solution of **3** at room temperature.

**^1^H NMR (500 MHz, C_6_D_6_):** δ (ppm) 0.95 (s, 18H; NC(C*H*_3_)_3_), 1.24 (d, ^3^*J*_H–H_ = 6.8 Hz, 12H; CH(C*H*_3_)_2_), 1.27 (d, ^3^*J*_H–H_ = 6.8 Hz, 12H; CH(C*H*_3_)_2_), 1.29–1.31 (m, 36H; CH(C*H*_3_)_2_), 1.37 (d, ^3^*J*_H–H_ = 6.8 Hz, 12H; CH(C*H*_3_)_2_), 2.74 (s, 6H; N(C*H*_3_)_2_), 2.89 (sept, ^3^*J*_H–H_ = 6.8 Hz, 4H; C*H*(CH_3_)_2_), 3.15–3.25 (m, 8H; C*H*(CH_3_)_2_), 4.72 (d, ^3^*J*_H–P_ = 3.7 Hz, 2H; NC*H*_2_Ph), 6.84–6.93 (m, 6H; Ar*H*), 7.02 (t, ^3^*J*_H–H_ = 7.8 Hz, 1H; Ar*H*), 7.06 (d, ^3^*J*_H–H_ = 7.4 Hz, 1H; Ar*H*), 7.18 (t, ^4^*J*_H–H_ = 1.5 Hz, 2H; Ar*H*), 7.20–7.22 (m, 8H; Ar*H*), 7.29–7.32 (m, 3H; Ar*H*), 7.38 (d, ^3^*J*_H–H_ = 7.8 Hz, 2H; Ar*H*).

**^13^C{^1^H} NMR (125 MHz, C_6_D_6_):** δ (ppm) 24.35, 24.41, 24.54, 24.78, 25.04, 25.12 (s; CH(*C*H_3_)_2_), 30.91, 30.96, 31.72, 34.94 (s; *C*H(CH_3_)_2_), 31.40 (s; NC(*C*H_3_)_3_), 40.34 (s; N*C*(CH_3_)_3_), 48.87 (br; N*C*H_2_Ph), 53.36 (s; N(*C*H_3_)_2_), 120.70, 120.84, 125.27, 129.42, 129.71, 129.80, 130.26, 130.42, 130.51, 133.20, 133.70, 137.92, 138.05, 139.45, 140.37, 147.10, 148.05 (s; Ar*C*), 173.10 (s; N*C*N).

**^31^P{^1^H} NMR (202 MHz, C_6_D_6_):** δ (ppm) 133.08 (s).

**^29^Si{^1^H} NMR (99.4 MHz, C_6_D_6_):** δ (ppm) –100.63 (d, ^3^*J*_Si–P_ = 8.4 Hz).

**HRMS (*m*/*z*):** [M+H]^+^ Calcd. for C_102_H_138_O_2_N_4_PSi, 1510.0321; Found 1510.0332.

**1.4. One-pot synthesis of 2**

**Scheme S3.** One-pot synthesis of **2**.

**1b** (144.8 mg, 0.34 mmol; 5 eq.) was dissolved in benzene (3 ml) and subsequently Me_3_SiCl (364.9 mg, 3.4 mmol; 5 eq.) was added to the mixture. **A** (80 mg, 0.067 mmol; 1 eq.) was dissolved in benzene (1 ml), to which the mixture of **1b** and Me_3_SiCl was added over 1 min. The reaction was further stirred for 1 min. at room temperature, before all volatiles were removed *in vacuo*. The residual was redissolved in benzene (1 ml) and MeCN (20 ml) was added before the mixture was left in a –35 °C freezer overnight to crystalize. The supernatant was removed, and the resulting solid was dried *in vacuo* to afford **2** (78 mg, 0.060 mmol; 89% yield) as a light-green powder. Single crystals of **2** suitable for X-ray diffraction were obtained by slow evaporation of a concentrated hexane solution.

**^1^H NMR (500 MHz, C_6_D_6_):** δ (ppm) 1.14 (d, ^3^*J*_H–H_ = 6.8 Hz, 12H; CH(C*H*_3_)_2_), 1.17 (d, ^3^*J*_H–H_ = 6.8 Hz, 12H; CH(C*H*_3_)_2_), 1.22 (d, ^3^*J*_H–H_ = 6.8 Hz, 12H; CH(C*H*_3_)_2_), 1.28–1.30 (m, 24H; CH(C*H*_3_)_2_), 1.32–1.34 (m, 12H; CH(C*H*_3_)_2_), 2.84–3.05 (m, 12H; C*H*(CH_3_)_2_), 5.44 (s, 2H; NC*H*_2_Ph), 6.63 (t, ^3^*J*_H–H_ = 7.5 Hz, 2H; Ar*H*), 6.72–6.76 (m, 3H; Ar*H*), 6.94 (t, ^3^*J*_H–H_ = 7.7 Hz, 1H; Ar*H*), 7.10 (s, 2H; Ar*H*), 7.16–7.17 (m, overlapping with C_6_D_6_, 8H; Ar*H*), 7.20 (d, ^4^*J*_H–H_ = 1.4 Hz, 4H; Ar*H*), 7.25 (d, ^3^*J*_H–H_ = 7.7 Hz, 2H; Ar*H*). (Accurate integration was not possible due to the presence of the minor isomer, **2**-*cis*; Ratio **2**-*trans*:**2**-*cis* = 1 : 0.38).

**^13^C{^1^H} NMR (125 MHz, C_6_D_6_):** δ (ppm) 24.24, 24.42, 24.45, 24.74, 25.10 (s; *C*H(CH_3_)_2_), 30.82, 30.89, 31.04, 34.88 (s; *C*H(CH_3_)_2_), 61.21 (br; N*C*H_2_Ph), 120.64, 120.71, 120.83, 129.28, 130.56, 131.24, 131.43, 131.81, 131.92, 132.53, 135.06, 137.08, 137.22, 139.22, 139.83, 140.98, 141.46, 141.60, 143.85, 146.75, 146.81, 146.92, 148.49, 148.58 (s; Ar*C*).

**^31^P{^1^H} NMR (202 MHz, C_6_D_6_):** δ (ppm) 633.92 (s, ^1^*J*_P–Te_ = 1747.8 Hz; **2**-*trans*), 624.95 (s, ^1^*J*_P–Te_ = 1760.3 Hz; **2**-*cis*).

**^125^Te NMR (157.7 MHz, *d*_8_-tol):** δ (ppm) Not detected.

**HRMS (*m*/*z*):** [M+H]^+^ Calcd. for C_85_H_109_NPTe, 1304.7354; Found 1304.7381.

**1.5. Synthesis of A from 2**

**Scheme S4.** Synthesis of **A** from **2**.

**2** (10 mg, 0.0077 mmol; 1 eq.) was dissolved in C_6_D_6_ (0.5 ml), and the solution was transferred to an NMR tube equipped with a J. Young gas-tight valve. The solution was degassed three times through freeze-pump-thaw cycles and subsequently charged with N_2_O (1 atm). The mixture was heated at 80 °C for 30 min, and the reaction process was monitored by ^31^P{^1^H} NMR spectroscopy. Once **2** was consumed completely, the mixture was filtered to remove elemental tellurium, and MeCN (20 ml) was added to the filtrate. The mixture was left in a freezer overnight at –35 °C to crystalize. The supernatant was removed, and the resulting solid was dried *in vacuo* to afford **A** (6.7 mg, 0.0056 mmol; 73% yield) as a white powder. All analytical data match literature reported values.^[1]^

**1.6. Synthesis of B from 2**

**Scheme S5.** Synthesis of **B** from **2**.

**2** (10 mg, 0.0077 mmol; 1 eq.) was dissolved in C_6_D_6_ (0.5 ml), and elemental sulfur (2.0 mg, 0.063 mmol; 8.1 eq.) was added to the solution. Immediately black precipitate was observed. The reaction was stirred for 30 min at room temperature. Once **2** was completely consumed, the mixture was filtered to remove elemental tellurium and MeCN (20 ml) was added to the filtrate. The mixture was left in a –35 °C freezer overnight to crystalize. The supernatant was removed, and the resulting solid was dried *in vacuo* to afford **B** (7.4 mg, 0.0061 mmol; 80% yield) as a light-green powder. All analytical data match literature reported values.^[52]^

**1.7. Synthesis of C from 2**

**Scheme S6.** Synthesis of **C** from **2**.

**2** (10 mg, 0.0077 mmol; 1 eq.) was dissolved in C_6_D_6_ (0.5 ml), and elemental selenium (2.0 mg, 0.025 mmol; 3.3 eq.) was added to the solution. The mixture was heated at 80 °C for 30 min, and the reaction process was monitored by ^31^P{^1^H} NMR spectroscopy. Once **2** was completely consumed, the mixture was filtered and MeCN (20 ml) was added to the filtrate. The mixture was left in a –35 °C freezer overnight to crystalize. The supernatant was removed and the resulting solid was dried *in vacuo* to afford **C** (6.9 mg, 0.0055 mmol; 71% yield) as a light-red powder. All analytical data match literature reported values.^[52]^

**1.8. Synthesis of 4 from 2**

**Scheme S7.** Synthesis of **4** from **2**.

**2** (10 mg, 0.0077 mmol; 1 eq.) was dissolved in C_6_D_6_ (0.5 ml), and AdN_3_ (2.0 mg, 0.011 mmol; 1.5 eq.) was added to the solution. A black precipitate was observed immediately, and the reaction was stirred for 30 min at room temperature. Once **2** was completely consumed, the mixture was filtered to remove elemental tellurium, and MeCN (20 ml) was added to the filtrate. The mixture was left in a –35 °C freezer overnight to crystalize. The supernatant was removed and the resulting solid was dried *in vacuo* to afford **4** (6.7 mg, 0.0051 mmol; 66% yield) as a white powder. Single crystals of **4** suitable for X-ray diffraction were obtained by slow evaporation of a concentrated hexane solution of **4** at room temperature.

**^1^H NMR (500 MHz, C_6_D_6_):** δ (ppm) 1.18 (d, ^3^*J*_H–H_ = 6.9 Hz, 18H; CH(C*H*_3_)_2_), 1.25 (d, ^3^*J*_H–H_ = 6.9 Hz, 18H; CH(C*H*_3_)_2_), 1.29–1.33 (m, 48H; CH(C*H*_3_)_2_), 1.49 (s, 12H; Ad C*H*_2_), 1.83 (s, 3H; Ad C*H*), 2.89 (sept, ^3^*J*_H–H_ = 6.8 Hz, 4H; C*H*(CH_3_)_2_), 3.09–3.16 (m, 8H; C*H*(CH_3_)_2_), 4.49 (br, 2H; NC*H*_2_Ph), 7.00–7.06 (m, 6H; Ar*H*), 7.16–7.18 (m, overlapping with C_6_D_6_, 8H; Ar*H*), 7.22 (d, ^4^*J*_H–H_ = 1.4 Hz, 4H; Ar*H*), 7.25 (t, ^4^*J*_H–H_ = 1.4 Hz, 2H; Ar*H*), 7.46 (d, ^3^*J*_H–H_ = 7.5 Hz, 2H; Ar*H*). Several resonances are broad due to fast rotation about the N–P bond, see VT-NMR for details.

**^13^C{^1^H} NMR (125 MHz, C_6_D_6_):** δ (ppm) 24.18, 24.42, 24.45, 24.49, 25.00, 25.42 (s; CH(*C*H_3_)_2_), 30.06, 30.52, 30.98, 31.06, 34.87, 36.70 (s; *C*H(CH_3_)_2_), 35.94 (s; Ad *C*H), 41.61 (s; Ad *C*H_2_), 48.27 (d, ^3^*J*_P–C_ = 6.5 Hz; Ad *C*H_2_), 57.82 (d, ^2^*J*_P–C_ = 14.6 Hz; Ad *C*), 120.68, 120.72, 129.78, 130.54, 131.13, 131.97, 136.99 (br), 137.47, 140.69, 141.10, 141.75, 146.85, 146.91, 148.37 (s; Ar*C*). Several resonances are broad or missing due to fast rotation about the N–P bond, see VT-NMR for details.

**^31^P{^1^H} NMR (202 MHz, C_6_D_6_):** δ (ppm) 316.36 (br).

**HRMS(*m*/*z*):** [M+H]^+^ Calcd. for C_95_H_124_N_2_P, 1323.9500; Found 1323.9509.

**1.9. Synthesis of 5a from 2**

**Scheme S8.** Synthesis of **5a** from **2**.

**2** (10 mg, 0.0077 mmol; 1 eq.) was dissolved in C_6_D_6_ (0.5 ml) and S_2_Ph_2_ (1.8 mg, 0.0077 mmol; 1 eq.) was added to the solution. A black precipitate was observed upon mixing, and the reaction was stirred for 30 min at room temperature. Once **2** was completely consumed, the mixture was filtered to remove elemental tellurium, and MeCN (20 ml) was added to the filtrate. The mixture was left in a –35 °C freezer overnight to crystalize. The supernatant was removed, and the resulting solid was dried *in vacuo* to afford **5a** (7.2 mg, 0.0052 mmol; 67% yield) as a white powder.

**^1^H NMR (500 MHz, C_6_D_6_):** δ (ppm) 1.19 (d, ^3^*J*_H–H_ = 6.9 Hz, 24H; CH(C*H*_3_)_2_), 1.25 (d, ^3^*J*_H–H_ = 6.9 Hz, 12H; CH(C*H*_3_)_2_), 1.31 (d, ^3^*J*_H–H_ = 6.9 Hz, 36H; CH(C*H*_3_)_2_), 2.90 (sept, ^3^*J*_H–H_ = 6.8 Hz, 4H; C*H*(CH_3_)_2_), 3.20 (br, 8H; C*H*(CH_3_)_2_), 4.70 (d, ^3^*J*_P–H_ = 10.2 Hz, 2H; NC*H*_2_Ph), 6.71 (t, ^3^*J*_H–H_ = 6.8 Hz, 2H; Ar*H*), 6.77 (t, ^3^*J*_P–H_ = 7.5 Hz, 1H; Ar*H*), 6.81–6.87 (m, 6H; Ar*H*), 7.00 (t, ^3^*J*_H–H_ = 7.7 Hz, 1H; Ar*H*), 7.16–7.19 (m, 8H; Ar*H*), 7.20 (d, ^4^*J*_H–H_ = 1.7 Hz, 4H; Ar*H*), 7.22–7.23 (m, 5H; Ar*H*), 7.31–7.34 (m, 4H; Ar*H*). Several resonances are broad or missing due to close contact between S–Ph and *^i^*Pr group, see VT-NMR for details.

**^13^C{^1^H} NMR (125 MHz, C_6_D_6_):** δ (ppm) 24.39, 24.42, 24.50, 25.25 (br) (s; CH(*C*H_3_)_2_), 30.91, 30.94, 34.91 (s; *C*H(CH_3_)_2_), 56.31 (d; ^2^*J*_P–C_ = 26.2 Hz; N*C*H_2_Ph), 120.72, 120.76, 126.24, 128.59, 128.92, 129.96, 130.77, 131.24, 132.94, 134.07, 134.20, 134.27, 134.32, 137.23, 137.63, 139.51 (d; ^4^*J*_P–C_ = 2.5 Hz), 141.14, 141.62, 142.20 (d; ^3^*J*_P–C_ = 3.9 Hz), 147.11, 148.31 (s; Ar*C*). Several resonances are broad or missing due to close contact between S–Ph and *^i^*Pr group, see VT-NMR for details.

**^31^P{^1^H} NMR (202 MHz, C_6_D_6_):** δ (ppm) 167.11 (br, **5a**)

**HRMS(*m*/*z*):** [M+H]^+^ Calcd. for C_97_H_119_NPS_2_, 1392.8516; Found 1392.8518.

**1.10. Synthesis of 5b from 2**

**Scheme S9.** Synthesis of **5b** from **2**.

**2** (10 mg, 0.0077 mmol; 1 eq.) was dissolved in C_6_D_6_ (0.5 ml), and Se_2_Ph_2_ (2.4 mg, 0.0077 mmol; 1 eq.) was added to the solution. A black precipitate was observed immediately, and the reaction was stirred for 30 min at room temperature. Once **2** was completely consumed, the mixture was filtered to remove elemental tellurium and MeCN (20 ml) was added to the filtrate. The mixture was left in a –35 °C freezer overnight to crystalize. The supernatant was removed, and the resulting solid was dried *in vacuo* to afford **5b** (7.7 mg, 0.0052 mmol; 67% yield) as a white powder.

**^1^H NMR (500 MHz, C_6_D_6_):** δ (ppm) 1.19–1.28 (m, 48H; CH(C*H*_3_)_2_), 1.33 (d, ^3^*J*_H–H_ = 6.9 Hz, 24H; CH(C*H*_3_)_2_), 2.91 (sept, ^3^*J*_H–H_ = 6.8 Hz, 4H; C*H*(CH_3_)_2_), 4.54 (d, ^3^*J*_P–H_ = 13.8 Hz, 2H; NC*H*_2_Ph), 6.71 (t, ^3^*J*_H–H_ = 6.8 Hz, 2H; Ar*H*), 6.76 (t, ^3^*J*_H–H_ = 7.3 Hz, 1H; Ar*H*), 6.82 (t, ^3^*J*_H–H_ = 7.4 Hz, 4H; Ar*H*), 6.89 (t, ^3^*J*_H–H_ = 7.1 Hz, 2H; Ar*H*), 7.01 (t, ^3^*J*_H–H_ = 7.4 Hz, 1H; Ar*H*), 7.16 (s, overlapping with C_6_D_6_, 4H; Ar*H*), 7.21–7.23 (m, 10H; Ar*H*), 7.30–7.34 (m, 8H; Ar*H*). Several resonances are broad or missing due to close contact between Se–Ph and *^i^*Pr group, see VT-NMR for details.

**^13^C{^1^H} NMR (125 MHz, C_6_D_6_):** δ (ppm) 24.37, 24.50, 24.53, 25.19 (br) (s; CH(*C*H_3_)_2_), 30.89, 30.97, 34.92 (s; *C*H(CH_3_)_2_), 58.08 (d; ^2^*J*_P–C_ = 38.4 Hz; N*C*H_2_Ph), 120.76, 120.80, 126.38, 128.59, 129.08, 129.41, 129.87, 130.37 (d; ^3^*J*_P–C_ = 18.5 Hz), 130.81, 131.50, 131.73, 132.89, 135.78, 135.81, 137.16 (d; ^4^*J*_P–C_ = 2.4 Hz), 137.59, 140.76 (d; ^3^*J*_P–C_ = 7.2 Hz), 141.23, 141.51, 142.08 (br), 147.12, 147.19, 148.32 (s; Ar*C*). Several resonances are broad or missing due to close contact between Se–Ph and *^i^*Pr group, see VT-NMR for details.

**^31^P{^1^H} NMR (202 MHz, C_6_D_6_):** δ (ppm) 191.52 (br, **5b**)

**HRMS(*m*/*z*):** [M+H]^+^ Calcd. for C_97_H_119_NP^78^Se^80^Se, 1486.7413; Found 1486.7448.

# **2. NMR spectra**

**Figure S1.** ^1^H NMR spectrum of **1b** (500 MHz, C_6_D_6_).

**Figure S2.** ^13^C{^1^H} NMR spectrum of **1b** (125 MHz, C_6_D_6_).

**Figure S3.** ^29^Si{^1^H} NMR spectrum of **1b** (99.4 MHz, C_6_D_6_).

**Figure S4.** *In-situ* ^31^P{^1^H} NMR spectrum of the reaction between **A** and **1b** (202 MHz, C_6_D_6_).

**Figure S5.** ^1^H NMR spectrum of **3** (500 MHz, C_6_D_6_).

**Figure S6.** ^13^C{^1^H} NMR spectrum of **3** (125 MHz, C_6_D_6_).

**Figure S7.** ^31^P{^1^H} NMR spectrum of **3** (202 MHz, C_6_D_6_).

**Figure S8.** ^29^Si{^1^H} NMR spectrum of **3** (99.4 MHz, C_6_D_6_).

**Figure S9.** ^1^H NMR spectrum of **2** (500 MHz, C_6_D_6_).

**Figure S10.** ^13^C{^1^H} NMR spectrum of **2** (125 MHz, C_6_D_6_).

**Figure S11.** ^31^P{^1^H} NMR spectrum of **2** (202 MHz, C_6_D_6_).

**Figure S12.** Zoomed in ^31^P{^1^H} NMR spectrum of **2** (202 MHz, C_6_D_6_).

**Figure S13.** ^1^H NMR spectrum of **4** (500 MHz, C_6_D_6_).

**Figure S14.** ^13^C{^1^H} NMR spectrum of **4** (125 MHz, C_6_D_6_).

**Figure S15.** ^31^P{^1^H} NMR spectrum of **4** (202 MHz, C_6_D_6_).

**Figure S16.** ^1^H NMR spectrum of **5a** (500 MHz, C_6_D_6_).

**Figure S17.** ^13^C{^1^H} NMR spectrum of **5a** (125 MHz, C_6_D_6_).

**Figure S18.** ^31^P{^1^H} NMR spectrum of **5a** (202 MHz, C_6_D_6_).

**Figure S19.** ^1^H NMR spectrum of **5b** (500 MHz, C_6_D_6_).

**Figure S20.** ^13^C{^1^H} NMR spectrum of **5b** (125 MHz, C_6_D_6_).

**Figure S21.** ^31^P{^1^H} NMR spectrum of **5b** (202 MHz, C_6_D_6_).

# **3. High Resolution Mass Spectra**


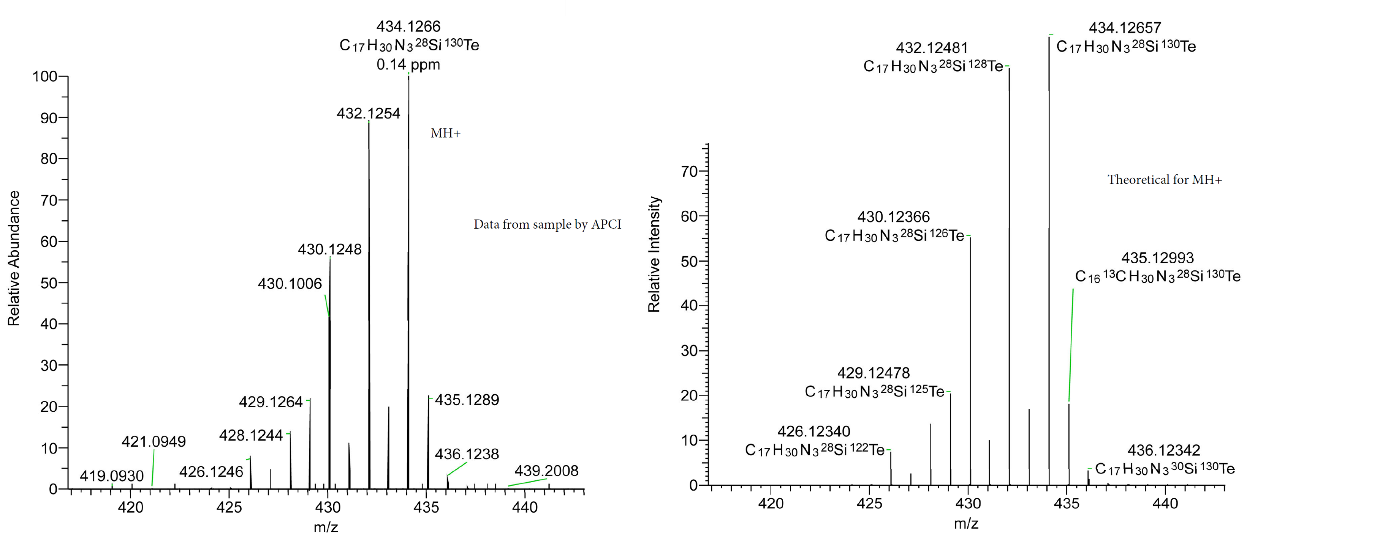


**Figure S22.** High resolution mass spectrum of **1b** (left: experimental; right: calculated).


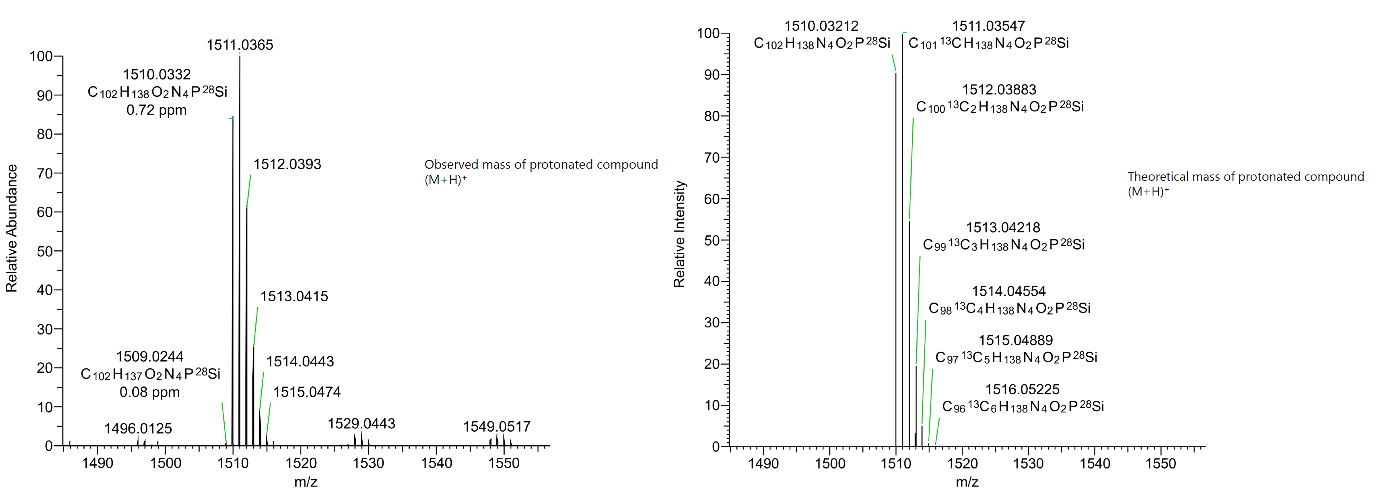


**Figure S23.** High resolution mass spectrum of **3** (left: experimental; right: calculated).


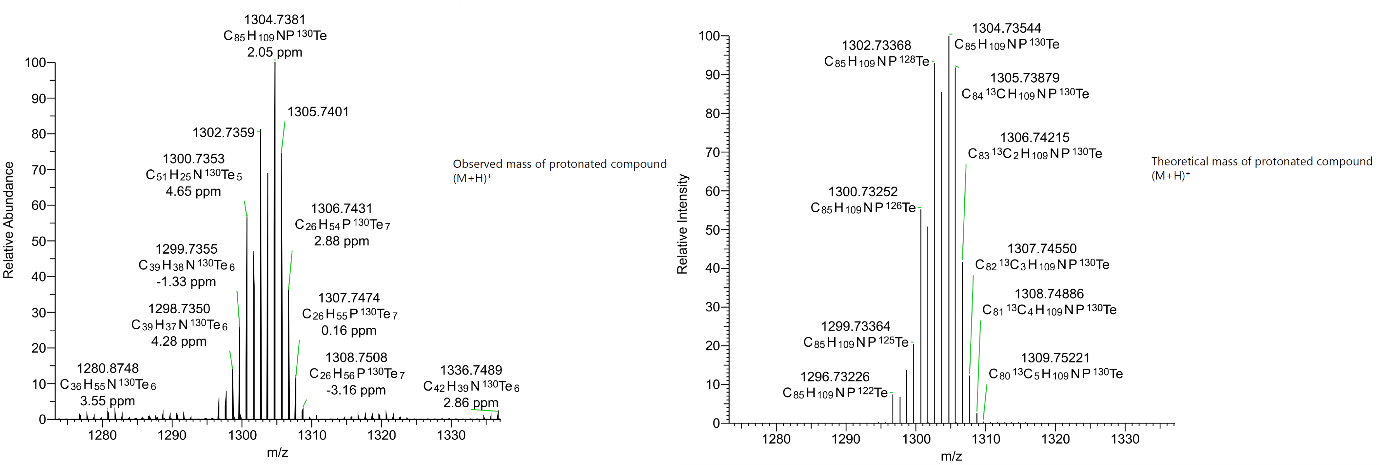


**Figure S24.** High resolution mass spectrum of **2** (left: experimental; right: calculated).


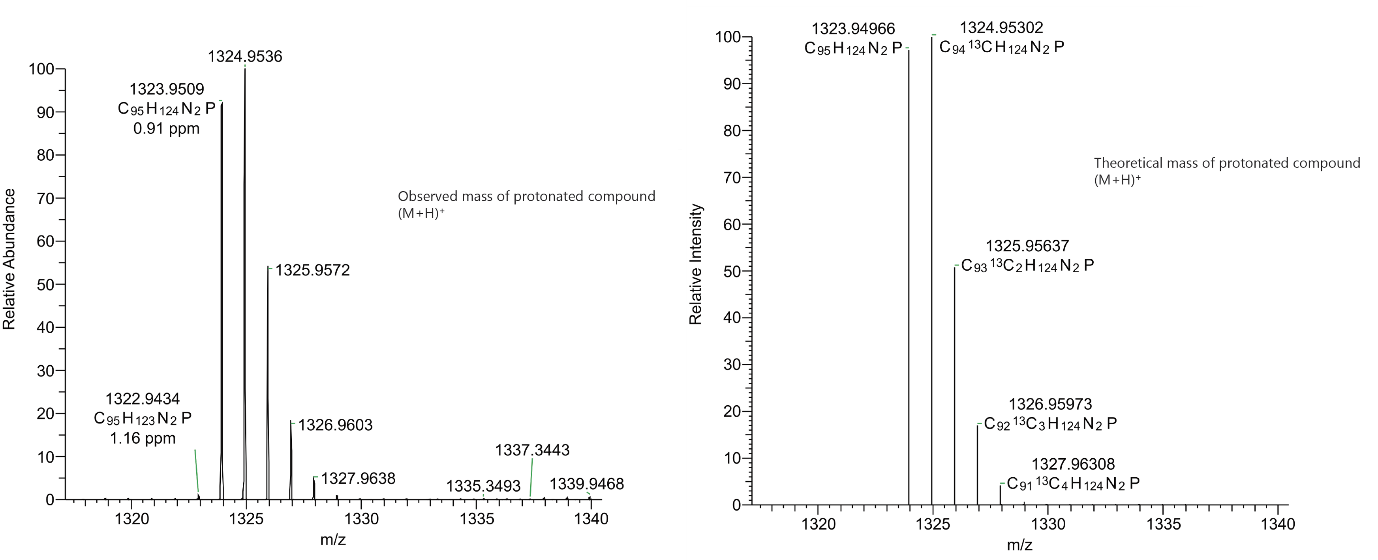


**Figure S25.** High resolution mass spectrum of **4** (left: experimental; right: calculated).


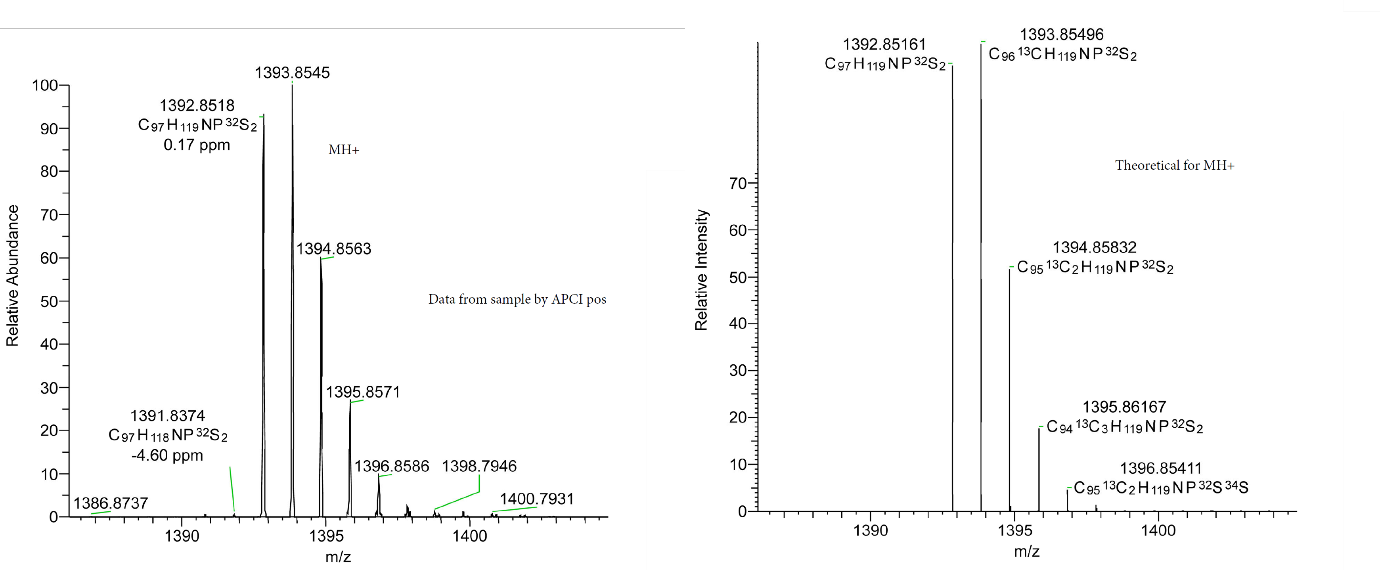


**Figure S26.** High resolution mass spectrum of **5a** (left: experimental; right: calculated).


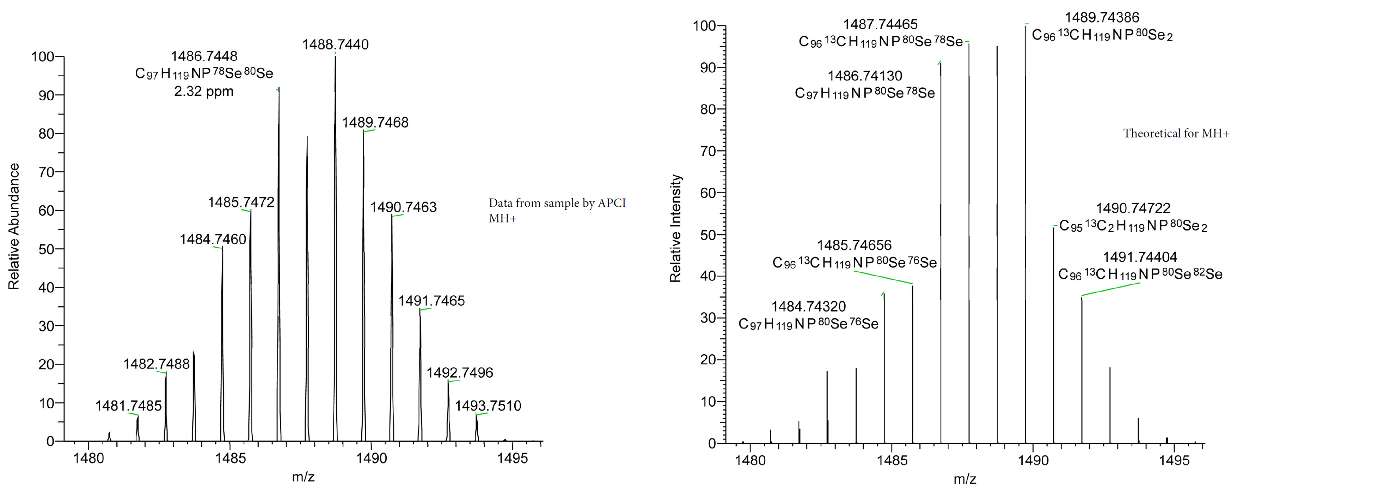


**Figure S27.** High resolution mass spectrum of **5b** (left: experimental; right: calculated).

# **4. Variable Temperature NMR studies of compound 2, 4, 5a and 5b**

**4.1. Variable Temperature ^31^P{^1^H}** **NMR of 2**

**Figure S28.** Variable-temperature ^31^P{^1^H} NMR spectrum of **2** in *d*_8_-toluene from 298K to 343K.

**4.2. Variable Temperature ^1^H** **NMR of 4**

**Figure S29.** Variable-temperature ^1^H NMR spectrum of **4** in *d*_8_-toluene from 298K to 343K.

**4.3. Variable Temperature ^31^P{^1^H}** **NMR of 5a**

**Figure S30.** Variable-temperature ^31^P{^1^H} NMR spectrum of **5a** in *d*_8_-toluene from 238K to 358K.

**4.4. Variable Temperature ^1^H NMR of 5a**

**Figure S31.** Variable-temperature ^1^H NMR spectrum of **5a** in *d*_8_-toluene from 238K to 358K.

**4.5. Variable Temperature ^1^H** **NMR of 5b**

**Figure S32.** Variable-temperature ^1^H NMR spectrum of **5b** in *d*_8_-toluene from 238K to 358K.

# **5. UV-vis Spectrum of 2**


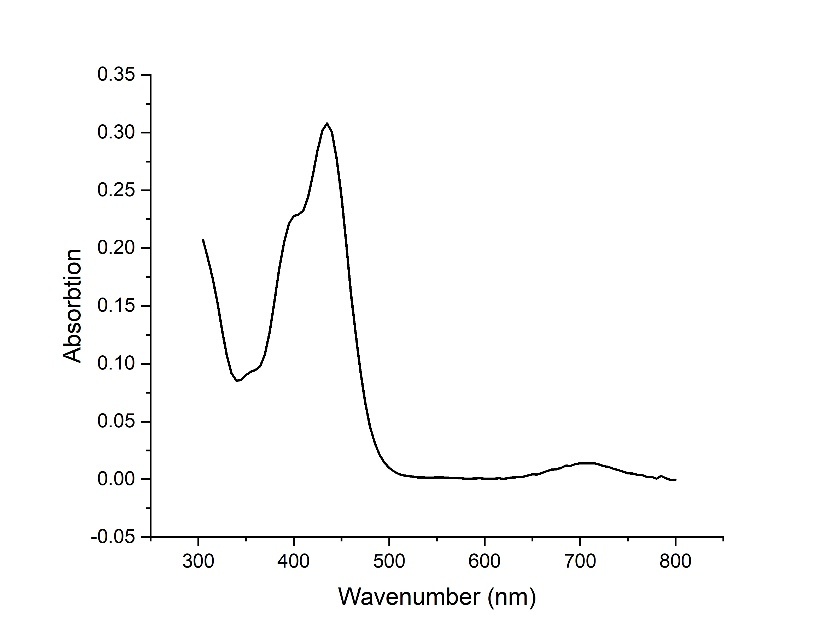

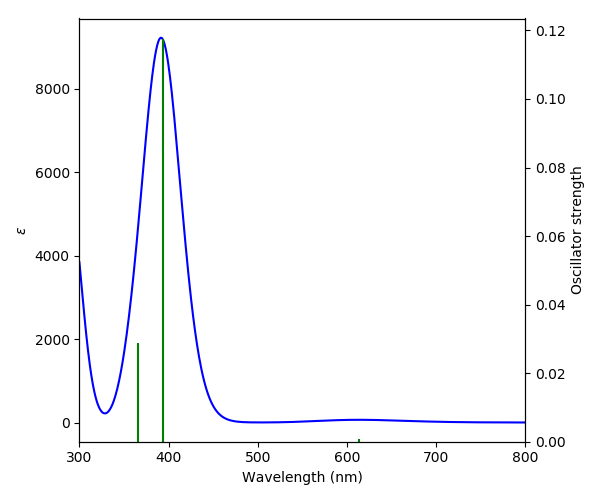


**Figure S33.**Experimental (left) and calculated (right) UV-Vis spectrum of **2** in n-hexane.

# **6. X-ray Crystallographic Data**

**Table S1.** Crystal data and structure refinement for **1b** (CCDC 2475938).

Empirical formula C_17_H_29_N_3_SiTe

Formula weight 431.12

Crystal color, shape, size yellow block, 0.077 × 0.125 × 0.167 mm^3^

Temperature 173(2) K

Wavelength 1.54178 Å (Cu *K*_α_)

Crystal system, space group Monoclinic, *P*2_1_/*n*

Unit cell dimensions *a* = 11.8969(8) Å α = 90°

*b* = 13.6317(8) Å β = 104.994(3)°

*c* = 13.3776(7) Å γ = 90°

Volume 2095.6(2) Å^3^

*Z* 4

Density (calculated) 1.366 mg/m^3^

Absorption coefficient 11.746 mm^–1^

*F*(000) 872

***Data collection***

Diffractometer Venture D8, Bruker

Source Iμ3.0, Incoatec

Detector Photon III

Theta range for data collection 4.44 to 68.62°

Index ranges –14 ≤ h ≤ 14, –16 ≤ k ≤ 16, –16 ≤ l ≤ 15

Reflections collected 33822

Independent reflections 3859 [*R*_int_ = 0.0783; *R*_sig_ = 0.0419]

Observed Reflections 3484

Completeness to theta = 67.679° 100%

***Solution and Refinement***

Absorption correction Multi-scan

Max. and min. transmission 0.753 and 0.525

Solution Intrinsic methods

Refinement method Full-matrix least-squares on *F*^2^

Weighting scheme w = [σ^2^*F_o_*^2^ + AP^2^ + BP]^–1^, with

P = (*F_o_*^2^ + 2 *F_c_*^2^)/3, A = 0.0595, B = 2.9701

Data / restraints / parameters 3859 / 0 / 207

Goodness-of-fit on *F*^2^ 1.104

Final *R* indices [I > 2σ(I)] *R*_1_ = 0.0443, w*R*_2_ = 0.1204

*R* indices (all data) *R*_1_ = 0.0480, w*R*_2_ = 0.1227

Largest diff. peak and hole 1.166 and –1.516 e·Å^–3^

Goodness-of-fit = [Σ[*w*(F_o_^2^ − F_c_^2^)^2^]/N_observns_ − N_params_)]^1/2^, all data. R1 = Σ(|F_o_| − |F_c_|) / Σ |F_o_|. *w*R2 = [Σ[*w*(F_o_^2^ − F_c_^2^)^2^] / Σ [*w*(F_o_^2^)^2^]]^1/2^.

**Crystal structure data for 1b**

A block, yellow-shaped specimen of C_17_H_29_N_3_SiTe (IUMSC 24371) of approximate dimensions 0.077 × 0.125 × 0.167 mm^3^, was placed on a Kapton mount with inert oil for crystal structure determination. The X-ray intensity data were measured on a Bruker D8 Venture KAPPA diffractometer equipped with a microfocus sealed tube (λ = 1.54178 Å) and a multilayer mirror monochromator.

**Data collection**

The data collection was performed using 1° ω and φ scans, frame times of 1 and 4 s, and a detector distance of 40.00 mm. Overall, 2706 frames were collected with a total exposure time of 1.39 hours. The frames were integrated with the SAINT V8.41 package using a narrow-frame algorithm.⁠^[79]^ The integration of the data using a monoclinic unit cell yielded 33822 reflections to a maximum θ angle of 68.62° (0.83 Å resolution), of which 3859 were independent (average redundancy 8.76, completeness = 100.0%, *R*_int_ = 7.83%, *R*_sig_ = 4.19%) and 3484 (90.3%) were greater than 2σ(*F*^2^). The final cell constants of *a* = 11.8969(8) Å, *b* = 13.6317(8) Å, *c* = 13.3776(7) Å, α = 90°, β = 104.994(3)°, γ = 90°, volume = 2095.6(2) Å^3^, are based upon the refinement of the XYZ-centroids of 9847 reflections above 20 σ(*I*) with 4.43° < 2θ < 68.40°. Data were corrected for absorption effects using the Multi-Scan method in SADABS 2016/2. The calculated minimum and maximum transmission coefficients (based on crystal size) are 0.525 and 0.753.⁠^[80]^ Additional crystal and refinement information can be found in the tables.

**Structure solution and refinement**

The space group *P*2_1_/*n* (14) was determined based on intensity statistics and systematic absences. The structure was solved by SHELXT 2018/2 and refined with full-matrix least squares / difference Fourier cycles using SHELXL-2019/2; *Z* = 4 for the formula unit C_17_H_29_N_3_SiTe.⁠^[81,82]^ Non-hydrogen atoms were refined with anisotropic displacement parameters. The hydrogen atoms were placed in ideal positions and refined as riding atoms with relative isotropic displacement parameters. The final anisotropic full-matrix least-squares refinement on *F*^2^ with 207 variables against 3859 data points and converged at *R*_1_ = 4.43%, for the observed data and w*R*_2_ = 12.27% for all data. The goodness-of-fit on *F*^2^ was 1.10. The largest peak in the final difference electron density synthesis was 1.17 e^−^/Å^3^ and the deepest hole was −1.52 e^−^/Å^3^ with an RMS deviation of 0.096 e^−^/Å^3^. On the basis of the final model, the calculated density was 1.37 g/cm^3^ and *F*(000), 872 e^−^.

**Table S2.** Crystal data and structure refinement for **2**·hex (CCDC 2475939).

Empirical formula C_91_H_122_NPTe

Formula weight 1388.46

Crystal color, shape, size red block, 0.258 × 0.367 × 0.568 mm^3^

Temperature 173(2) K

Wavelength 1.54178 Å (Cu *K*_α_)

Crystal system, space group Monoclinic, *P*2_1_/*n*

Unit cell dimensions *a* = 22.4048(6) Å α = 90°

*b* = 15.6232(4) Å β = 94.2950(10)°

*c* = 23.7447(6) Å γ = 90°

Volume 8288.1(4) Å^3^

*Z* 4

Density (calculated) 1.113 mg/m^3^

Absorption coefficient 3.321 mm^–1^

*F*(000) 2968

***Data collection***

Diffractometer Venture D8, Bruker

Source Iμ3.0, Incoatec

Detector Photon III

Theta range for data collection 2.615 to 68.81°

Index ranges –26 ≤ h ≤ 27, –18 ≤ k ≤ 18, –28 ≤ l ≤ 28

Reflections collected 174691

Independent reflections 15257 [*R*_int_ = 0.0751; *R*_sig_ = 0.0447]

Observed Reflections 13864

Completeness to theta = 67.679° 100%

***Solution and Refinement***

Absorption correction Multi-scan

Max. and min. transmission 0.753 and 0.424

Solution Intrinsic methods

Refinement method Full-matrix least-squares on *F*^2^

Weighting scheme w = [σ^2^*F_o_*^2^ + AP^2^ + BP]^–1^, with

P = (*F_o_*^2^ + 2 *F_c_*^2^)/3, A = 0.0962, B = 6.9413

Data / restraints / parameters 15257 / 0 / 793

Goodness-of-fit on *F*^2^ 1.028

Final *R* indices [I > 2σ(I)] *R*_1_ = 0.0564, w*R*_2_ = 0.1580

*R* indices (all data) *R*_1_ = 0.0612, w*R*_2_ = 0.1657

Largest diff. peak and hole 0.711 and –0.608 e·Å^–3^

Goodness-of-fit = [Σ[*w*(F_o_^2^ − F_c_^2^)^2^]/N_observns_ − N_params_)]^1/2^, all data. R1 = Σ(|F_o_| − |F_c_|) / Σ |F_o_|. *w*R2 = [Σ[*w*(F_o_^2^ − F_c_^2^)^2^] / Σ [*w*(F_o_^2^)^2^]]^1/2^.

**Crystal structure data for 2**

A red, block-shaped specimen of C_85_H_108_NPTe (IUMSC s24373) approximate dimensions 0.258 × 0.367 × 0.568 mm^3^, was placed on a Kapton mount with inert oil for crystal structure determination. The X-ray intensity data were measured on a Bruker D8 Venture KAPPA diffractometer equipped with a microfocus sealed tube (λ = 1.54178 Å) and a multilayer mirror monochromator.

**Data collection**

The data collection was performed using 1° ω and φ scans, frame times of 0.75, 1, 2, 5, 7 and 10 s, and a detector distance of 40.00 mm. Overall, 3696 frames were collected with a total exposure time of 6.12 hours. The frames were integrated with the SAINT V8.41 package using a narrow-frame algorithm.⁠^[79]^ The integration of the data using a monoclinic unit cell yielded 174691 reflections to a maximum θ angle of 68.81° (0.83 Å resolution), of which 15257 were independent (average redundancy 11.45, completeness = 100.0%, *R*_int_ = 7.51%, *R*_sig_ = 4.47%) and 13864 (90.9%) were greater than 2σ(*F*^2^). The final cell constants of *a* = 22.4048(6) Å, *b* = 15.6232(4) Å, *c*= 23.7447(6) Å, α = 90°, β = 94.2950(10)°, γ = 90°, volume = 8288.1(4) Å^3^, are based upon the refinement of the XYZ-centroids of 9442 reflections above 20 σ(*I*) with 3.73° < 2θ < 68.39°. Data were corrected for absorption effects using the Multi-Scan method in SADABS 2016/2. The calculated minimum and maximum transmission coefficients (based on crystal size) are 0.424 and 0.753.⁠^[80]^ Additional crystal and refinement information can be found in the tables.

**Structure solution and refinement**

The space group *P*2_1_/*n* (14) was determined based on intensity statistics and systematic absences. The structure was solved by XT, VERSION 2014/5 and refined with full-matrix least squares / difference Fourier cycles using SHELXL-2019/1; Z = 4 for the formula unit C_85_H_108_NPTe.⁠^[81,82]^ Non-hydrogen atoms were refined with anisotropic displacement parameters. The hydrogen atoms were placed in ideal positions and refined as riding atoms with relative isotropic displacement parameters. Remaining electron density indicated that additional partial solvent (hexane) was present in the structure. However, solvent models with strong sets of restraints and constraints did not converge to a chemically sensible structure. Therefore, the structure was investigated for solvent accessible areas.^[83]^ Two voids were found in the unit cell (~1000 Å^3^) to contain 184 electrons. For comparison, hexane occupies ca. 163 Å^3^ with 50 electrons.^[84]^ Based on these values, we estimate that there is one molecule of hexane per formula unit. The contribution of the unidentified solvent to the structure factors was assessed by back-Fourier transformation^[84]^ and the data were corrected accordingly. The final anisotropic full-matrix least-squares refinement on *F*^2^ with 793 variables against 15257 data points and converged at *R*_1_ = 5.64%, for the observed data and w*R*_2_ = 16.57% for all data. The goodness-of-fit on *F*^2^ was 1.03. The largest peak in the final difference electron density synthesis was 0.71 e^−^/Å^3^ and the deepest hole was −0.61 e^−^/Å^3^ with an RMS deviation of 0.074 e^−^/Å^3^. On the basis of the final model, the calculated density was 1.04 g/cm^3^ and *F*(000), 2768 e^−^.

**Table S3.** Crystal data and structure refinement for **3**·hex (CCDC 2475940).

Empirical formula C_108_H_151_N_4_O_2_PSi

Formula weight 1596.38

Crystal color, shape, size colorless plate, 0.108 × 0.111 × 0.258 mm^3^

Temperature 173(2) K

Wavelength 1.54178 Å (Cu *K*_α_)

Crystal system, space group Monoclinic, *C*2/*c*

Unit cell dimensions *a* = 33.7055(9) Å α = 90°

*b* = 17.3627(5) Å β = 113.9060(10)°

*c* = 36.9832(13) Å γ = 90°

Volume 19786.5(11) Å^3^

*Z* 8

Density (calculated) 1.072 mg/m^3^

Absorption coefficient 0.726 mm^–1^

*F*(000) 6976

***Data collection***

Diffractometer Venture D8, Bruker

Source Iμ3.0, Incoatec

Detector Photon III

Theta range for data collection 2.615 to 68.885°

Index ranges –40 ≤ h ≤ 40, –20 ≤ k ≤ 20, –44 ≤ l ≤ 44

Reflections collected 110684

Independent reflections 18148 [*R*_int_ = 0.1020; *R*_sig_ = 0.0761]

Observed Reflections 13650

Completeness to theta = 67.679° 100%

***Solution and Refinement***

Absorption correction Multi-scan

Max. and min. transmission 0.753 and 0.562

Solution Intrinsic methods

Refinement method Full-matrix least-squares on *F*^2^

Weighting scheme w = [σ^2^*F_o_*^2^ + AP^2^ + BP]^–1^, with

P = (*F_o_*^2^ + 2 *F_c_*^2^)/3, A = 0.0762, B = 17. 7597

Data / restraints / parameters 18148 / 0 / 991

Goodness-of-fit on *F*^2^ 1.043

Final *R* indices [I > 2σ(I)] *R*_1_ = 0.0615, w*R*_2_ = 0.1582

*R* indices (all data) *R*_1_ = 0.0819, w*R*_2_ = 0.1720

Largest diff. peak and hole 0.665 and –0.528 e·Å^–3^

Goodness-of-fit = [Σ[*w*(F_o_^2^ − F_c_^2^)^2^]/N_observns_ − N_params_)]^1/2^, all data. R1 = Σ(|F_o_| − |F_c_|) / Σ |F_o_|. *w*R2 = [Σ[*w*(F_o_^2^ − F_c_^2^)^2^] / Σ [*w*(F_o_^2^)^2^]]^1/2^.

**Crystal structure data for 3**

A colorless, plate-shaped specimen of C_102_H_137_N_4_O_2_PSi (IUMSC 24377_sq) approximate dimensions 0.108 × 0.111 × 0.258 mm^3^, was placed on a Kapton mount with inert oil for crystal structure determination. The X-ray intensity data were measured on a Bruker D8 Venture KAPPA diffractometer equipped with a microfocus sealed tube (λ = 1.54178 Å) and a multilayer mirror monochromator.

**Data collection**

The data collection was performed using 1° ω and φ scans, frame times of 0.75, 1, 20, 62 and 90 s, and a detector distance of DISTANCE mm. Overall, 2192 frames were collected with a total exposure time of 26.17 hours. The frames were integrated with the SAINT V8.41 package using a narrow-frame algorithm.⁠^[79]^ The integration of the data using a monoclinic unit cell yielded 110684 reflections to a maximum θ angle of 68.88° (0.83 Å resolution), of which 18148 were independent (average redundancy 6.10, completeness = 100.0%, *R*_int_ = 10.20%, *R*_sig_ = 7.61%) and 13650 (75.2%) were greater than 2σ(*F*^2^). The final cell constants of *a*= 33.7055(9) Å, *b* = 17.3627(5) Å, *c* = 36.9832(13) Å, α = 90°, β = 113.9060(10)°, γ = 90°, volume = 19786.5(11) Å^3^, are based upon the refinement of the XYZ-centroids of 9903 reflections above 20 σ(*I*) with 2.61° < 2θ < 68.39°. Data were corrected for absorption effects using the Multi-Scan method in SADABS 2016/2. The calculated minimum and maximum transmission coefficients (based on crystal size) are 0.562 and 0.753.⁠^[80]^ Additional crystal and refinement information can be found in the tables.

**Structure solution and refinement**

The space group *C*2/*c* (15) was determined based on intensity statistics and systematic absences. The structure was solved by XT, VERSION 2014/5 and refined with full-matrix least squares / difference Fourier cycles using SHELXL-2019/1; *Z* = 8 for the formula unit C_102_H_137_N_4_O_2_PSi.⁠^[81,82]^ Non-hydrogen atoms were refined with anisotropic displacement parameters. The hydrogen atoms were placed in ideal positions and refined as riding atoms with relative isotropic displacement parameters. Remaining electron density indicated that additional partial solvent (hexane) was present in the structure. However, solvent models with strong sets of restraints and constraints did not converge to a chemically sensible structure. Therefore, the structure was investigated for solvent accessible areas.^[83]^ Two voids were found in the unit cell (~1920 Å^3^) to contain 408 electrons. For comparison, hexane occupies ca. 163 Å^3^ with 50 electrons.^[84]^ Based on these values, we estimate that there is one molecule of hexane per formula unit. The contribution of the unidentified solvent to the structure factors was assessed by back-Fourier transformation^[84]^ and the data were corrected accordingly. The final anisotropic full-matrix least-squares refinement on *F*^2^ with 991 variables against 18148 data points and converged at *R*_1_ = 6.15%, for the observed data and w*R*_2_ = 17.20% for all data. The goodness-of-fit on *F*^2^ was 1.04. The largest peak in the final difference electron density synthesis was 0.67 e^−^/Å^3^ and the deepest hole was −0.53 e^−^/Å^3^ with an RMS deviation of 0.050 e^−^/Å^3^. On the basis of the final model, the calculated density was 1.01 g/cm^3^ and *F*(000), 6576 e^−^.

**Table S4.** Crystal data and structure refinement for **4**·0.5hex (CCDC 2475941).

Empirical formula C_98_H_129_N_12_P

Formula weight 1365.99

Crystal color, shape, size colorless block, 0.109 × 0.192 × 0.66 mm^3^

Temperature 173(2) K

Wavelength 0.71073 Å (Mo *K*_α_)

Crystal system, space group Triclinic, *P*–1

Unit cell dimensions *a* = 12.6644(7) Å α = 99.790(2)°

*b* = 19.0601(11) Å β = 93.436(2)°

*c* = 19.5443(10) Å γ = 109.078(2)°

Volume 4359.7(4) Å^3^

*Z* 2

Density (calculated) 1.041 mg/m^3^

Absorption coefficient 0.077 mm^–1^

*F*(000) 1492

***Data collection***

Diffractometer Venture D8, Bruker

Source Iμ3.0, Incoatec

Detector Photon III

Theta range for data collection 2.00 to 26.43°

Index ranges –16 ≤ h ≤ 16, –24 ≤ k ≤ 24, –25 ≤ l ≤ 25

Reflections collected 162745

Independent reflections 17898 [*R*_int_ = 0.0952; *R*_sig_ = 0.0557]

Observed Reflections 14410

Completeness to theta = 25.242° 99.9%

***Solution and Refinement***

Absorption correction Multi-scan

Max. and min. transmission 0.745 and 0.538

Solution Intrinsic methods

Refinement method Full-matrix least-squares on *F*^2^

Weighting scheme w = [σ^2^*F_o_*^2^ + AP^2^ + BP]^–1^, with

P = (*F_o_*^2^ + 2 *F_c_*^2^)/3, A = 0.0908, B = 2.7100

Data / restraints / parameters 17898 / 151 / 1017

Goodness-of-fit on *F*^2^ 1.026

Final *R* indices [I > 2σ(I)] *R*_1_ = 0.0673, w*R*_2_ = 0.1754

*R* indices (all data) *R*_1_ = 0.0818, w*R*_2_ = 0.1904

Largest diff. peak and hole 0.877 and −0.341 e·Å^–3^

Goodness-of-fit = [Σ[*w*(F_o_^2^ − F_c_^2^)^2^]/N_observns_ − N_params_)]^1/2^, all data. R1 = Σ(|F_o_| − |F_c_|) / Σ |F_o_|. *w*R2 = [Σ[*w*(F_o_^2^ − F_c_^2^)^2^] / Σ [*w*(F_o_^2^)^2^]]^1/2^.

**Crystal structure data for 4**

A colorless, block-shaped specimen of C_85_H_104_N_12_P (IUMSC 25004_sq) approximate dimensions 0.109 × 0.192 × 0.668 mm^3^, was placed on a Kapton mount with inert oil for crystal structure determination. The X-ray intensity data were measured on a Bruker D8 Venture KAPPA diffractometer equipped with a microfocus sealed tube (λ = 0.71073 Å) and a multilayer mirror monochromator.

**Data collection**

The data collection was performed using 1° ω and φ scans, frame times of 1, 10 and 30 s, and a detector distance of 40.00 mm. Overall, 1565 frames were collected with a total exposure time of 4.28 hours. The frames were integrated with the SAINT V8.41 package using a narrow-frame algorithm.⁠^[79]^ The integration of the data using a triclinic unit cell yielded 162745 reflections to a maximum θ angle of 26.43° (0.80 Å resolution), of which 20114 were independent (average redundancy 8.09, completeness = 99.9%, *R_i_*_nt_ = 9.57%, *R*_sig_ = 5.57%) and 14410 (71.6%) were greater than 2σ(*F*^2^). The final cell constants of *a* = 12.6644(7) Å, *b* = 19.0601(11) Å, *c* = 19.5443(10) Å, α = 99.790(2)°, β = 93.436(2)°, γ = 109.078(2)°, volume = 4359.7(4) Å^3^, are based upon the refinement of the XYZ-centroids of 9104 reflections above 20 σ(*I*) with 2.36° < 2θ < 26.41°. Data were corrected for absorption effects using the Multi-Scan method in SADABS 2016/2. The calculated minimum and maximum transmission coefficients (based on crystal size) are 0.538 and 0.745.⁠^[80]^ Additional crystal and refinement information can be found in the tables.

**Structure solution and refinement**

The space group *P*–1 (2) was determined based on intensity statistics and systematic absences. The structure was solved by SHELXT 2018/2 and refined with full-matrix least squares / difference Fourier cycles using SHELXL-2019/2; *Z* = 2 for the formula unit C_85_H_104_N_12_P.⁠^[81,82]^ Non-hydrogen atoms were refined with anisotropic displacement parameters. The hydrogen atoms were placed in ideal positions and refined as riding atoms with relative isotropic displacement parameters. Remaining electron density indicated that additional partial solvent (hexane) was present in the structure. However, solvent models with strong sets of restraints and constraints did not converge to a chemically sensible structure. Therefore, the structure was investigated for solvent accessible areas.^[83]^ One void was found in the unit cell (~254 Å^3^) to contain 48 electrons. For comparison, hexane occupies ca. 163 Å^3^ with 50 electrons.^[84]^ Based on these values, we estimate that there is half a molecule of hexane per formula unit. The contribution of the unidentified solvent to the structure factors was assessed by back-Fourier transformation^[84]^ and the data were corrected accordingly. The final anisotropic full-matrix least-squares refinement on *F*^2^ with 1017 variables against 17898 data points and 151 restraints converged at *R*_1_ = 6.73%, for the observed data and w*R*_2_ = 19.04% for all data. The goodness-of-fit on *F*^2^ was 1.03. The largest peak in the final difference electron density synthesis was 0.88 e^−^/Å^3^ and the deepest hole was −0.34 e^−^/Å^3^ with an RMS deviation of 0.061 e^−^/Å^3^. On the basis of the final model, the calculated density was 1.01 g/cm^3^ and *F*(000), 1442 e^−^.

# **7. Computational Details**

Geometry optimizations were carried out using the Gaussian 16 package with the M06-2X functional.^[85,86]^ The def2-SVP basis set was used for all the atoms.^[87,88]^ Frequency calculations at the same level of theory were performed to identify the number of imaginary frequencies (zero for local minimum and one for transition states) and provide the thermal corrections of Gibbs free energy. Single-point energy calculations were performed at the M06-2X/def2-TZVP level of theory for the modelling of compounds in solution (benzene). The gas-phase geometry was used for all the solution phase calculations. The SMD method was used with the corresponding solvent, while Bondi radii were chosen as the atomic radii to define the molecular cavity.^[89,90]^ The corrections of Gibbs free energy from frequency calculations were added to the single-point energies to obtain the Gibbs free energy in solution. All the energies reported in the paper correspond to the reference state of 1 mol/L, 298K. Natural bond orbital (NBO) calculations were carried out using NBO 7.0 program at the M06-2X/def2-SVP level of theory.^[91]^ Optimized structures and orbitals were visualized using Chemcraft.^[92]^ The UV-Vis absorption spectrum was calculated using gas-phase geometry with TD-DFT at the M06-2X/def2-SVP level of theory, and the PCM method was used with the solvent of n-hexane.^[93–99]^ The UV-Vis absorption spectrum was drawn with GaussSum package.^[100]^ Intrinsic bond orbitals (IBOs) were carried out using ORCA 6.0.1 program at the M06-2X/def2-TZVP level of theory, and visualized by IBOview program.^[101,102]^

**7.1 Energy Calculation for *cis-trans* isomerization of 2**

**Table S5**. Energies of Intermediates and Transition States.

| **Species** | **Thermal Corrections of Gibbs Free Energies (Hartree)** | **Solvation Energies (Hartree)** |
| --- | --- | --- |
| **2-*trans*** | 1.582756 | –3968.042884 |
| ***ts*-2**  **2-*cis*** | 1.582156  1.58117 | –3968.008568  –3968.041704 |

**7.2 Cartesian coordinates for computed structures**

**2**

C 0.16100000 0.15678700 1.56382400

C -1.06032800 -0.00793300 2.25137000

C -1.04481300 -0.11688900 3.64681200

C 0.15046700 -0.09772800 4.35678700

C 1.35270800 0.05132900 3.67259600

C 1.37390300 0.19959100 2.28320600

C 2.68061800 0.32922400 1.58440400

C 3.00446100 1.46118200 0.82947700

C 4.20958900 1.51895200 0.12207200

C 5.04910000 0.39761300 0.12310900

C 4.75344500 -0.74050100 0.88148400

C 3.58045800 -0.73958100 1.64273100

C 5.64680400 -1.94070300 0.86131000

C 6.93387600 -1.87399400 1.44217300

C 7.75293600 -3.00372800 1.39354300

C 7.33545600 -4.19396500 0.79411900

C 6.05859100 -4.23984400 0.23770500

C 5.19819800 -3.13691200 0.25706400

C 4.65309000 2.78705600 -0.53873800

C 4.15958600 3.17354300 -1.80287900

C 4.61371000 4.37231200 -2.35993400

C 5.53703900 5.19250400 -1.70892300

C 6.00636000 4.79069100 -0.45937800

C 5.57914900 3.60362900 0.14250600

C -3.27750200 0.99166400 1.67055200

C -4.54299900 0.93379800 1.07992500

C -4.90621300 -0.21906000 0.37773800

C -4.03063800 -1.30166500 0.25022900

C -2.76781500 -1.22327600 0.84785000

C -2.38356700 -0.08019400 1.55811800

C -4.45999200 -2.55001400 -0.45643300

C -4.99286000 -3.61205100 0.29739800

C -5.35205700 -4.79480600 -0.36059400

C -5.21897100 -4.93966100 -1.73924500

C -4.70883800 -3.86210200 -2.47023400

C -4.31364300 -2.67238200 -1.85678900

C -5.48971300 2.08836500 1.16300800

C -5.44715900 3.08697100 0.17267600

C -6.35616000 4.14866400 0.25072600

C -7.29648200 4.24173200 1.27453000

C -7.31326400 3.24014200 2.25043400

C -6.42499800 2.16428600 2.21726600

C 7.39801500 -0.62340500 2.18133700

C 3.83211900 -3.22627000 -0.41809400

C 3.20488400 2.27458900 -2.57759800

C 6.11667600 3.25285700 1.52429100

C -5.14417000 -3.51966600 1.80993500

C -3.71454600 -1.54292900 -2.68241800

C -4.40154100 3.07218000 -0.93305200

C -6.49874300 1.07355600 3.27743200

C 8.91703000 -0.49149000 2.27954400

C 6.76457000 -0.56509200 3.57720500

C 3.85450600 -2.54066300 -1.78894400

C 3.28570400 -4.64712700 -0.54860400

C 3.98011700 1.13728600 -3.25460000

C 2.36596100 3.01893700 -3.61383300

C 7.63640300 3.06331800 1.50192400

C 5.70369100 4.30259000 2.56064500

C -3.97464100 -4.22100900 2.51006500

C -6.48499700 -4.06679800 2.30538500

C -3.01437200 -2.02395900 -3.95475600

C -4.75768800 -0.48113900 -3.05298200

C -3.28709500 4.07574900 -0.61518000

C -4.98986400 3.33352800 -2.32057100

C -6.82633000 1.60932200 4.67237900

C -7.50424900 -0.00982600 2.86820400

H -1.99545200 -0.24013100 4.16838600

H 0.14545900 -0.19802300 5.44276300

H 2.30029000 0.07654800 4.21403000

H 2.32408800 2.31646400 0.80964400

H 5.97187000 0.42410000 -0.46391300

H 3.33828500 -1.61531200 2.25155100

H 8.74820200 -2.95965400 1.83950200

H 5.72793700 -5.16968000 -0.22827200

H 4.24000300 4.68756700 -3.33535800

H 6.72324000 5.42395500 0.07161400

H -2.97931000 1.88877000 2.21930800

H -5.89886100 -0.27559300 -0.07538200

H -2.08994000 -2.07404500 0.77517600

H -5.75078400 -5.63148800 0.21883500

H -4.59551400 -3.96430300 -3.55173600

H -6.32761600 4.93299800 -0.51041000

H -8.04203500 3.30301300 3.06213000

H 7.03362900 0.25110900 1.62373800

H 3.11720600 -2.67463300 0.20761900

H 2.51003200 1.82055800 -1.84979800

H 5.67159500 2.29790700 1.83796000

H -5.10589400 -2.45516000 2.08519600

H -2.95697200 -1.05388300 -2.04804000

H -3.94426200 2.07102500 -0.96112500

H -5.50603800 0.60091200 3.33065000

H 9.34764300 -1.24427000 2.95702600

H 9.39810100 -0.59982100 1.29665200

H 9.18085900 0.49643100 2.68322900

H 7.08804900 -1.42891700 4.17831800

H 7.06755800 0.35392500 4.10230700

H 5.66685900 -0.58140600 3.51936400

H 2.85582500 -2.58033200 -2.25276100

H 4.15491500 -1.48672600 -1.70889800

H 4.56547900 -3.04819100 -2.45910500

H 3.30784500 -5.18170800 0.41246000

H 2.24124500 -4.60706400 -0.89316900

H 3.85439100 -5.23649700 -1.28400700

H 3.28936100 0.43516900 -3.74623300

H 4.65925100 1.54715100 -4.01814300

H 4.58702300 0.57513000 -2.53227300

H 1.81889500 3.86296900 -3.16930900

H 2.99003200 3.40193800 -4.43532400

H 1.63008700 2.33385900 -4.05756200

H 7.93295500 2.29942100 0.76728100

H 8.15160400 3.99936300 1.23665600

H 7.99996700 2.74978500 2.49267200

H 6.05479700 4.01478000 3.56291200

H 6.13307200 5.28796700 2.32321700

H 4.61019800 4.41089700 2.59532200

H -3.01170900 -3.78121900 2.21255800

H -4.07006100 -4.14235100 3.60370800

H -3.95388200 -5.29000300 2.24407100

H -6.60135100 -3.86458200 3.38038400

H -7.32757200 -3.60127900 1.77418500

H -6.55684200 -5.15634400 2.16959700

H -3.74068500 -2.32826000 -4.72405200

H -2.41261100 -1.20632000 -4.37953300

H -2.34692000 -2.87821000 -3.76778000

H -5.55167300 -0.92538200 -3.67352300

H -5.22498100 -0.03601600 -2.16552700

H -4.28428800 0.33273400 -3.62487500

H -2.50826400 4.04567000 -1.39319500

H -3.69145300 5.09938100 -0.56754100

H -2.81696500 3.85255200 0.35429700

H -5.81776000 2.64413100 -2.54141100

H -5.36794400 4.36296500 -2.41621500

H -4.20956800 3.19514500 -3.08437300

H -6.15992300 2.43770600 4.95214800

H -7.86385500 1.97024300 4.73566800

H -6.71710600 0.80927700 5.41897800

H -7.54480100 -0.81036800 3.62269700

H -8.51209800 0.42260100 2.76999800

H -7.23517500 -0.46204000 1.90325600

N 0.16407700 0.24969400 0.12829900

P -0.73054700 1.53315300 -0.49706600

C -8.27307800 5.40172200 1.33562600

H -8.06143500 6.04610100 0.46697100

C -8.06993100 6.23874400 2.60180200

H -7.03562300 6.60317800 2.67420200

H -8.74593000 7.10672600 2.60800700

H -8.28097400 5.64229600 3.50275900

C -9.72232300 4.92057100 1.22098000

H -9.87442200 4.33619400 0.30272300

H -9.99080800 4.28117000 2.07602600

H -10.41664400 5.77389300 1.20945300

C -5.59139200 -6.23842200 -2.42965900

H -5.99860100 -6.90962100 -1.65606900

C -4.35493700 -6.91238800 -3.03297400

H -4.61673400 -7.87961800 -3.48722800

H -3.91189800 -6.28072800 -3.81875600

H -3.58553300 -7.08660400 -2.26681700

C -6.67643900 -6.02947500 -3.48915700

H -7.56914900 -5.55820600 -3.05479600

H -6.31327700 -5.38038100 -4.30053700

H -6.97293500 -6.98959600 -3.93682100

C 8.24828700 -5.40535400 0.75325600

H 7.69683000 -6.20620600 0.23448100

C 8.58315000 -5.90277600 2.16237400

H 7.66998700 -6.12756300 2.73112600

H 9.19827300 -6.81374900 2.11787200

H 9.15010900 -5.14228800 2.72106900

C 9.52260900 -5.11852200 -0.04605900

H 9.28451200 -4.78269400 -1.06503700

H 10.11870500 -4.32981300 0.43826400

H 10.15048300 -6.01926800 -0.11448400

C 6.00996200 6.48874300 -2.34028000

H 5.50548900 6.57423000 -3.31633900

C 7.52029200 6.47097900 -2.59193000

H 7.84142300 7.39254700 -3.09968800

H 8.07412400 6.40058600 -1.64305400

H 7.80881000 5.61252000 -3.21453100

C 5.60702000 7.70104500 -1.49573500

H 5.90681500 8.63719500 -1.98970700

H 4.52062900 7.72683900 -1.33200300

H 6.09600300 7.66998700 -0.50981800

C 0.85081200 -0.81823000 -0.62405800

H 1.90698900 -0.83617900 -0.32000500

H 0.81886800 -0.53887400 -1.68446800

C 0.22112100 -2.17586200 -0.42869300

C 0.41402000 -2.89547800 0.75847600

C -0.60483700 -2.71296400 -1.42112800

C -0.23443700 -4.11300600 0.95995900

H 1.06420800 -2.49143700 1.53825500

C -1.25089400 -3.93450900 -1.22340500

H -0.75220400 -2.15220400 -2.34771800

C -1.07536800 -4.63044200 -0.02764100

H -0.08569400 -4.65897900 1.89300100

H -1.91066300 -4.33790200 -1.99386700

H -1.59586800 -5.57605500 0.13340000

Te -1.01783800 1.31289800 -2.78085700

**2-*cis***

C 0.06225500 -0.18634500 -1.40409300

C -1.19935600 -0.09099500 -2.03203000

C -1.26547600 -0.20955100 -3.42603300

C -0.12274700 -0.39274000 -4.19571400

C 1.11647400 -0.48257500 -3.57178600

C 1.22069300 -0.40354500 -2.18043500

C 2.56124600 -0.49317300 -1.54024500

C 2.86352400 -1.51663000 -0.63787500

C 4.07917300 -1.51845600 0.05338900

C 4.97220900 -0.45844900 -0.14384600

C 4.70833100 0.55797400 -1.07010500

C 3.50783100 0.50726100 -1.78642100

C 5.66492300 1.69308000 -1.26223400

C 6.91633600 1.46506300 -1.87869400

C 7.79638600 2.53832900 -2.03122300

C 7.47399700 3.82611900 -1.59746100

C 6.23164000 4.02899200 -0.99949400

C 5.31410600 2.98839300 -0.82019700

C 4.43709800 -2.65596000 0.95674300

C 3.87360800 -2.75670100 2.24555800

C 4.23430600 -3.83705200 3.05508300

C 5.13404800 -4.81463000 2.62741200

C 5.67371500 -4.69623400 1.34642400

C 5.33808700 -3.63790800 0.49776000

C -3.57346600 -0.70254800 -1.53567700

C -4.78855600 -0.50161800 -0.87038800

C -4.88626700 0.52685200 0.07051600

C -3.80045000 1.37225700 0.32928300

C -2.61280900 1.17391000 -0.37511800

C -2.47323400 0.12643800 -1.29065200

C -3.88726000 2.47441900 1.33936000

C -4.59383100 3.65145800 1.02854600

C -4.63797800 4.68449200 1.97341900

C -4.00487200 4.57960700 3.20889400

C -3.30357800 3.40326100 3.49533100

C -3.22647000 2.34731000 2.58602800

C -5.96791900 -1.37582200 -1.15963000

C -6.14136700 -2.58290000 -0.45797900

C -7.26382800 -3.37001500 -0.73949900

C -8.20897000 -2.99567200 -1.69288300

C -8.01215600 -1.79455000 -2.38003700

C -6.90809200 -0.97670900 -2.13339000

C 7.27513000 0.09381200 -2.44085100

C 3.99132900 3.25301300 -0.10738600

C 2.95658900 -1.66823400 2.78360600

C 5.94970600 -3.59053200 -0.89609300

C -5.22523100 3.84781000 -0.34329800

C -2.43759000 1.08721700 2.92754800

C -5.17187000 -3.01811300 0.63037800

C -6.76601100 0.34527400 -2.87498200

C 8.77831700 -0.14457600 -2.57552100

C 6.57596900 -0.12758400 -3.78817200

C 4.05318800 2.78191400 1.35009800

C 3.51822200 4.70423100 -0.17132300

C 3.79222200 -0.55717000 3.43044600

C 1.89082600 -2.18110600 3.75069600

C 7.46208100 -3.35677000 -0.82754000

C 5.62122200 -4.85329300 -1.69815000

C -4.18444100 4.41079700 -1.31862600

C -6.48006800 4.72048500 -0.32414600

C -1.49737100 1.23431500 4.12315900

C -3.34486500 -0.12866200 3.15507600

C -4.72633200 -4.47329600 0.47571900

C -5.78047900 -2.77080200 2.01503400

C -7.02367100 0.21538000 -4.37772800

C -7.68234900 1.40551700 -2.25302300

H -2.24173200 -0.12667700 -3.90642700

H -0.19895900 -0.46639000 -5.28115100

H 2.02520800 -0.63771800 -4.15633400

H 2.14260500 -2.32151500 -0.47008600

H 5.90542500 -0.43312200 0.42675000

H 3.28455900 1.29611500 -2.51053100

H 8.76442200 2.36999500 -2.50656900

H 5.97518500 5.03457300 -0.66135900

H 3.80530800 -3.92562600 4.05536100

H 6.37395100 -5.45725200 0.98965400

H -3.48152400 -1.53309200 -2.24031900

H -5.82343100 0.67023300 0.61631300

H -1.78498500 1.85746300 -0.20513500

H -5.18156200 5.60276500 1.74093500

H -2.80271800 3.31308600 4.46095500

H -7.40990000 -4.30651700 -0.19443500

H -8.74460500 -1.48503000 -3.13043200

H 6.88883500 -0.66504900 -1.74514400

H 3.22165900 2.65170800 -0.60974700

H 2.43236500 -1.22480300 1.92141400

H 5.50720000 -2.74117700 -1.43635900

H -5.52226100 2.85850700 -0.71915300

H -1.81198600 0.87075100 2.04523600

H -4.26950700 -2.39303200 0.55142200

H -5.72895400 0.68957500 -2.74873500

H 9.21890800 0.47608500 -3.37034500

H 9.30955200 0.07181700 -1.63726600

H 8.96795600 -1.19395700 -2.84270500

H 6.92026200 0.61746100 -4.52203400

H 6.80196500 -1.13046800 -4.18182300

H 5.48487700 -0.03417700 -3.69298300

H 3.08021300 2.93951300 1.84341100

H 4.30726000 1.71489200 1.41915700

H 4.81566900 3.35068000 1.90453500

H 3.51194100 5.08477100 -1.20332900

H 2.49316300 4.77291700 0.22451100

H 4.15279600 5.36703500 0.43669800

H 3.15380000 0.28031100 3.75246100

H 4.32254200 -0.94638100 4.31341400

H 4.54610700 -0.16891900 2.73061000

H 1.31511900 -3.01050000 3.31314400

H 2.33330000 -2.52642500 4.69708400

H 1.18725800 -1.37174100 3.99592900

H 7.69961500 -2.43157400 -0.28098300

H 7.96646800 -4.18803700 -0.31078900

H 7.88841000 -3.27961300 -1.83944800

H 6.01341900 -4.77004200 -2.72268100

H 6.07001900 -5.74805400 -1.24064300

H 4.53503600 -5.01168400 -1.75566400

H -3.30453300 3.75432400 -1.38550200

H -4.61411500 4.52209700 -2.32595300

H -3.84195500 5.40092000 -0.97766600

H -6.96855200 4.69548800 -1.30905800

H -7.20362800 4.37131600 0.42683300

H -6.24343000 5.77325000 -0.10794000

H -2.05949100 1.30609300 5.06695400

H -0.85389600 0.34436200 4.19048600

H -0.85040300 2.12136000 4.05454700

H -4.00607900 0.04755400 4.01785600

H -3.97045400 -0.34817600 2.28122000

H -2.73562000 -1.02234500 3.36378200

H -3.95809800 -4.71491100 1.22525600

H -5.56371300 -5.17340500 0.61845900

H -4.30004400 -4.65307900 -0.52275600

H -6.07279200 -1.71720000 2.13663700

H -6.68112900 -3.38805400 2.16022000

H -5.05841700 -3.02215400 2.80662500

H -6.39554700 -0.56916700 -4.82332300

H -8.07490400 -0.02995100 -4.59105100

H -6.80178400 1.16595500 -4.88475600

H -7.54804400 2.37913900 -2.74913200

H -8.73843500 1.11039000 -2.35504400

H -7.47362800 1.53234400 -1.18039900

N 0.18047700 -0.06969500 0.02123000

P -0.33118700 -1.21837700 1.15085300

C -9.41695000 -3.86787200 -1.98077500

H -9.34946400 -4.74138100 -1.31214400

C -9.40522200 -4.37953800 -3.42414800

H -8.47644900 -4.92447600 -3.64373800

H -10.25554800 -5.05352900 -3.60608500

H -9.48120700 -3.54280500 -4.13561000

C -10.72525000 -3.13658100 -1.66710400

H -10.74332000 -2.78517300 -0.62593200

H -10.84999400 -2.25982200 -2.32112400

H -11.58945500 -3.79858000 -1.82602600

C -4.06206900 5.71303500 4.21642700

H -4.69413500 6.50214000 3.77772200

C -2.67251700 6.30807400 4.46351800

H -2.72680100 7.15064000 5.16883900

H -1.99514700 5.55352200 4.89275000

H -2.22410000 6.67180800 3.52770200

C -4.71107400 5.26860600 5.53014600

H -5.71299100 4.85212800 5.35592200

H -4.10531200 4.49433400 6.02534200

H -4.80279500 6.11646900 6.22499100

C 8.45049700 4.97369300 -1.77628100

H 7.97115600 5.87238600 -1.35531700

C 8.73351200 5.24267400 -3.25711500

H 7.80371400 5.43826700 -3.80946400

H 9.39770400 6.11167200 -3.37527300

H 9.22761900 4.37685100 -3.72415800

C 9.74986200 4.72872600 -1.00378200

H 9.55052600 4.55675800 0.06321600

H 10.27680100 3.84486200 -1.39502600

H 10.42671100 5.59099800 -1.09670900

C 5.50591400 -5.97960600 3.52577500

H 4.96600300 -5.83816900 4.47606100

C 7.00551900 -5.99432400 3.83488400

H 7.25160300 -6.81049500 4.53024100

H 7.59288600 -6.14825800 2.91657500

H 7.32757000 -5.04499200 4.28536600

C 5.05462000 -7.31343900 2.92353000

H 5.27874200 -8.14553800 3.60740900

H 3.97464700 -7.31122700 2.72030800

H 5.57591500 -7.50908800 1.97375300

C 0.96188900 1.06248300 0.58376400

H 2.01851700 0.94950900 0.29403500

H 0.92262700 0.95266200 1.67886600

C 0.45417700 2.43319600 0.20325100

C 0.57265900 2.91750500 -1.10667600

C -0.19378600 3.22484700 1.15680500

C 0.02797500 4.15229300 -1.45362000

H 1.08003300 2.31782000 -1.86527400

C -0.74997900 4.45662300 0.81138300

H -0.29903500 2.85595500 2.17847500

C -0.64006000 4.92097500 -0.49825800

H 0.12133300 4.51474900 -2.47836000

H -1.28802300 5.03532500 1.56532400

H -1.07830500 5.88007100 -0.77807400

Te -1.11927600 -3.11427500 0.12034300

***ts*-2**

C -0.12765200 -0.00376700 -1.65316600

C 1.09885600 -0.25848600 -2.30837600

C 1.13370200 -0.36973100 -3.70302400

C -0.02847600 -0.29031900 -4.46171000

C -1.24211400 -0.06526200 -3.82051700

C -1.30588800 0.11133500 -2.43551500

C -2.61905300 0.37766800 -1.79333200

C -2.77511100 1.48021800 -0.95233700

C -3.92264100 1.63999800 -0.17122100

C -4.94138600 0.68475100 -0.27356700

C -4.83017800 -0.40673400 -1.14508800

C -3.66720100 -0.54009200 -1.91276500

C -5.89049000 -1.46268100 -1.19629700

C -7.11843800 -1.20439700 -1.84304200

C -8.09160700 -2.20556000 -1.86716000

C -7.88067300 -3.45321500 -1.27593700

C -6.65776700 -3.68870600 -0.64894700

C -5.65259700 -2.71766700 -0.59335900

C -3.95242400 2.77434400 0.80686600

C -3.72151100 2.52762900 2.17444900

C -3.60412800 3.61632400 3.04836500

C -3.69424600 4.93233300 2.60534900

C -3.93746300 5.15418600 1.24569200

C -4.07398100 4.10341500 0.33864400

C 3.42633200 0.47302000 -1.76913900

C 4.64070300 0.33244500 -1.08847300

C 4.78544600 -0.72062200 -0.18331600

C 3.74962400 -1.63534400 0.04461600

C 2.55226500 -1.48804800 -0.65873000

C 2.37620500 -0.42673400 -1.55743000

C 3.94902000 -2.78375900 0.98495800

C 4.43424800 -4.00359700 0.47936800

C 4.58572100 -5.08600000 1.35509400

C 4.29488700 -4.98092600 2.71272300

C 3.83387300 -3.75206700 3.19656600

C 3.64130900 -2.65266800 2.35875300

C 5.76048900 1.30001200 -1.30298500

C 5.85671500 2.44274300 -0.48899500

C 6.91788900 3.33133100 -0.70100100

C 7.87755500 3.11361700 -1.68675900

C 7.75889300 1.97054200 -2.48393500

C 6.71645100 1.05881400 -2.31386900

C -7.34991500 0.11202800 -2.57317300

C -4.35977000 -2.99516200 0.16675700

C -3.60994600 1.11825800 2.73927600

C -4.40989900 4.37902500 -1.12155200

C 4.74737600 -4.18267100 -1.00011800

C 3.09534100 -1.34470300 2.91567300

C 4.81127700 2.76383800 0.56983800

C 6.64570800 -0.19716000 -3.17207200

C -8.82114200 0.51253500 -2.67568300

C -6.70930600 0.05825100 -3.96599400

C -4.42661100 -2.38477900 1.57137000

C -3.97841300 -4.47207700 0.24672000

C -4.93399500 0.70056000 3.39118700

C -2.45975100 0.94658500 3.73575200

C -5.90011200 4.11059600 -1.37047600

C -4.03524800 5.78290800 -1.59232400

C 3.56982300 -4.85030500 -1.71921600

C 6.04647300 -4.95372100 -1.24406500

C 2.27299300 -1.51934200 4.19462600

C 4.20666400 -0.31835300 3.17125100

C 3.88299900 3.87859000 0.07548400

C 5.43215600 3.12482100 1.92116900

C 7.09738500 0.02971600 -4.61574200

C 7.45217100 -1.33234200 -2.52887600

H 2.09504400 -0.55992900 -4.18410600

H 0.01121300 -0.40232700 -5.54565800

H -2.16608600 0.01437800 -4.39704500

H -1.95993600 2.19749000 -0.85409700

H -5.83605700 0.77815500 0.34860800

H -3.55397400 -1.41402300 -2.56116300

H -9.04323300 -2.01070700 -2.36604800

H -6.48886600 -4.66306900 -0.18644100

H -3.41937500 3.43200700 4.10982400

H -4.01569900 6.18174200 0.88590200

H 3.29521800 1.30154100 -2.47017200

H 5.73020300 -0.83628500 0.35423000

H 1.74843300 -2.20941400 -0.50581900

H 4.94561200 -6.04278400 0.96826500

H 3.59818200 -3.65864500 4.25883000

H 6.99702500 4.22744300 -0.07958100

H 8.50313600 1.78812600 -3.26278600

H -6.83466400 0.90069900 -2.00620200

H -3.54536200 -2.48578000 -0.36721500

H -3.41403000 0.43651800 1.89951500

H -3.83989700 3.66544200 -1.73411700

H 4.87637000 -3.18151300 -1.43752600

H 2.43294700 -0.92541600 2.14021600

H 4.19302900 1.86629700 0.72374600

H 5.59254900 -0.51557000 -3.20240100

H -9.37367800 -0.14175200 -3.36689700

H -9.32039200 0.47539800 -1.69675900

H -8.90295400 1.53750000 -3.06522500

H -7.17746800 -0.73564600 -4.56829200

H -6.84104800 1.01554300 -4.49249900

H -5.63253500 -0.15306600 -3.90066000

H -3.45691400 -2.48959200 2.08580900

H -4.68452400 -1.31794000 1.52874400

H -5.19580300 -2.89308600 2.17401500

H -3.97221300 -4.94405600 -0.74691500

H -2.97053100 -4.56830400 0.67783000

H -4.66869300 -5.03721200 0.89138200

H -4.86665200 -0.32036700 3.79625000

H -5.18191400 1.38346200 4.21869700

H -5.76584300 0.73180000 2.67317900

H -1.50669300 1.31151600 3.32443300

H -2.64802400 1.48370600 4.67745800

H -2.34051600 -0.11856800 3.98823200

H -6.17043300 3.08069600 -1.09687600

H -6.51508100 4.79457400 -0.76563500

H -6.15300300 4.26304500 -2.43068400

H -4.17556000 5.86206800 -2.67972600

H -4.67127100 6.55062900 -1.12603000

H -2.98635200 6.02038000 -1.36318500

H 2.64879600 -4.26041700 -1.60558200

H 3.78174500 -4.96243700 -2.79331800

H 3.38290200 -5.85205400 -1.30004400

H 6.29331100 -4.94696900 -2.31598000

H 6.88716800 -4.50614900 -0.69446400

H 5.96075500 -6.00696200 -0.93721400

H 2.92058200 -1.71435900 5.06322400

H 1.71237700 -0.59686500 4.40761000

H 1.55770300 -2.35166100 4.12282100

H 4.91320800 -0.70240400 3.92344800

H 4.77045900 -0.08776300 2.25895700

H 3.77746800 0.62251000 3.54907300

H 3.11815900 4.11180800 0.83142900

H 4.45543700 4.79590600 -0.13440600

H 3.36883600 3.58181400 -0.85114800

H 6.11893000 2.33913400 2.26843800

H 5.99429300 4.06972800 1.87140500

H 4.64230300 3.25190100 2.67629800

H 6.58103400 0.88788900 -5.06914200

H 8.18083400 0.21121300 -4.67904400

H 6.88395300 -0.86262300 -5.22191700

H 7.38337500 -2.25081700 -3.13170500

H 8.51351300 -1.04955800 -2.45117400

H 7.08797500 -1.55888100 -1.51673800

N -0.18191900 0.12056900 -0.23713100

P 0.95092500 1.09800900 0.66018300

C 9.01898000 4.09151100 -1.89604900

H 8.89145100 4.89767200 -1.15544800

C 8.96723500 4.72218200 -3.29054400

H 8.00099300 5.21521200 -3.46699400

H 9.76608900 5.46903300 -3.41057600

H 9.10273400 3.95729300 -4.07065100

C 10.37673400 3.43067000 -1.64135800

H 10.42266600 2.99483500 -0.63350600

H 10.56054900 2.62321300 -2.36675500

H 11.19159600 4.16302600 -1.74133000

C 4.44245200 -6.17412800 3.63848000

H 4.87882800 -6.99205100 3.04241900

C 3.07534200 -6.64244900 4.14753100

H 3.17648600 -7.53534600 4.78234900

H 2.59357200 -5.85494600 4.74823200

H 2.40440700 -6.88746400 3.31129400

C 5.39317100 -5.88244200 4.80213000

H 6.37667300 -5.55435100 4.43775300

H 4.99120300 -5.08873500 5.45016700

H 5.53389300 -6.77969500 5.42276000

C -8.95483700 -4.52435300 -1.31690100

H -8.55300000 -5.40329900 -0.78710400

C -9.27057300 -4.94692700 -2.75457600

H -8.36524000 -5.28936500 -3.27512700

H -10.00898700 -5.76233800 -2.76790400

H -9.69020700 -4.10484900 -3.32617600

C -10.22245000 -4.07392100 -0.58527300

H -10.00128200 -3.79055100 0.45329200

H -10.67253500 -3.20178100 -1.08399000

H -10.97284000 -4.87835500 -0.57423900

C -3.50869400 6.09445100 3.56255800

H -3.38825000 5.66493900 4.57043100

C -4.73143000 7.01525600 3.58797600

H -4.59986000 7.81451200 4.33226600

H -4.88422600 7.49390400 2.60861600

H -5.64459900 6.45642000 3.83643600

C -2.23473100 6.87769100 3.23034100

H -2.07228300 7.68788800 3.95673500

H -1.35287900 6.22104600 3.23919900

H -2.30701200 7.33073300 2.22945600

C -1.05754300 -0.76951000 0.54966600

H -2.10031700 -0.68823900 0.20939500

H -1.04908800 -0.40839400 1.58987600

C -0.60224200 -2.20825600 0.50694200

C -0.79217100 -2.98215100 -0.64623100

C 0.09766100 -2.75920400 1.58306200

C -0.26118800 -4.26891100 -0.72545800

H -1.34150400 -2.56478400 -1.49411000

C 0.63055900 -4.04676800 1.50827800

H 0.24341800 -2.15744700 2.48290400

C 0.45789200 -4.80031900 0.34789700

H -0.40418900 -4.85916300 -1.63218500

H 1.19981000 -4.45286900 2.34698900

H 0.88672300 -5.80147400 0.27944600

Te -0.07568300 3.09283500 1.19112700

# **8. References**

[78] R. Azhakar, R. S. Ghadwal, H. W. Roesky, H. Wolf, D. Stalke, “Facile Access to the Functionalized N-Donor Stabilized Silylenes PhC(N*^t^*Bu)_2_SiX (X = PPh_2_, NPh_2_, NCy_2_, N*^i^*Pr_2_, NMe_2_, N(SiMe_3_)_2_, O*^t^*Bu)” *Organometallics* **2012**, *31*, 4588–4592.

[79] SAINT V8.41 (2024), Bruker AXS, Madison, WI, USA.

[80] L. Krause, R. Herbst-Irmer, G. M. Sheldrick, D. Stalke, “Comparison of Ag and Mo microfocus X-ray sources for single-crystal structure determination” *J. Appl. Cryst.* **2015**, *48*, 3–10.

[81] G. M. Sheldrick, “SHELXT–Integrated space-group and crystal-structure determination” *Acta Cryst.* **2015**, *A71*, 3–8.

[82] G. M. Sheldrick, “Crystal structure refinement with SHELXL” *Acta Cryst.* **2015**, *C71*, 3–8.

[83] A. L. Spek, “PLATON SQUEEZE: a tool for the calculation of the disordered solvent contribution to the calculated structure factors” *Acta Cryst.* **2015**, *C71*, 9–18.

[84] A. Immirzi, B. Perini, “Prediction of density in organic crystals” *Acta Cryst.* **1977**, *A33*, 216–218.

[85] Gaussian 16, Revision C.01, M. J. Frisch, G. W. Trucks, H. B. Schlegel, G. E. Scuseria, M. A. Robb, J. R. Cheeseman, G. Scalmani, V. Barone, G. A. Petersson, H. Nakatsuji, X. Li, M. Caricato, A. V. Marenich, J. Bloino, B. G. Janesko, R. Gomperts, B. Mennucci, H. P. Hratchian, J. V. Ortiz, A. F. Izmaylov, J. L. Sonnenberg, D. Williams-Young, F. Ding, F. Lipparini, F. Egidi, J. Goings, B. Peng, A. Petrone, T. Henderson, D. Ranasinghe, V. G. Zakrzewski, J. Gao, N. Rega, G. Zheng, W. Liang, M. Hada, M. Ehara, K. Toyota, R. Fukuda, J. Hasegawa, M. Ishida, T. Nakajima, Y. Honda, O. Kitao, H. Nakai, T. Vreven, K. Throssell, J. A. Montgomery, Jr., J. E. Peralta, F. Ogliaro, M. J. Bearpark, J. J. Heyd, E. N. Brothers, K. N. Kudin, V. N. Staroverov, T. A. Keith, R. Kobayashi, J. Normand, K. Raghavachari, A. P. Rendell, J. C. Burant, S. S. Iyengar, J. Tomasi, M. Cossi, J. M. Millam, M. Klene, C. Adamo, R. Cammi, J. W. Ochterski, R. L. Martin, K. Morokuma, O. Farkas, J. B. Foresman, and D. J. Fox, Gaussian, Inc., Wallingford CT, 2016.

[86] Y. Zhao, D. G. Truhlar, “The M06 suite of density functionals for main group thermochemistry, thermochemical kinetics, noncovalent interactions, excited states, and transition elements: two new functionals and systematic testing of four M06-class functionals and 12 other functionals” *Theor. Chem. Acc.* **2008**, *120*, 215–241.

[87] F. Weigend, R. Ahlrichs, “Balanced basis sets of split valence, triple zeta valence and quadruple zeta valence quality for H to Rn: Design and assessment of accuracy” *Phys. Chem. Chem. Phys.* **2005**, *7*, 3297–3305.

[88] F. Weigend, “Accurate Coulomb-fitting basis sets for H to Rn” *Phys. Chem. Chem. Phys.* **2006**, *8*, 1057–1065.

[89] A. V. Marenich, C. J. Cramer, D. G. Truhlar, “Universal Solvation Model Based on Solute Electron Density and on a Continuum Model of the Solvent Defined by the Bulk Dielectric Constant and Atomic Surface Tensions” *J. Phys. Chem. B* **2009**, *113*, 6378–6396.

[90] A. Bondi, “van der Waals Volumes and Radii” *J. Phys. Chem* **1964**, *68*, 441–451.

[91] NBO 7.0. E. D. Glendening, J. K. Badenhoop, A. E. Reed, J. E. Carpenter, J. A. Bohmann, C. M. Morales, P. Karafiloglou, C. R. Landis, F. Weinhold, University of Wisconsin, Madison WI, 2018.

[92] ChemCraft - Graphical Software for Visualization of Quantum Chemistry Computations, Version 1.8. <http://www.chemcraftprog.com>.

[93] R. Bauernschmitt, R. Ahlrichs, “Treatment of electronic excitations within the adiabatic approximation of time dependent density functional theory” *Chem. Phys. Lett.* **1996**, *256*, 454–464.

[94] M. E. Casida, C. Jamorski, K. C. Casida, D. R. Salahub, “Molecular excitation energies to high-lying bound states from time-dependent density-functional response theory: Characterization and correction of the time-dependent local density approximation ionization threshold” *J. Chem. Phys.* **1998**, *108*, 4439–4449.

[95] R. E. Stratmann, G. E. Scuseria, M. J. Frisch, “An efficient implementation of time-dependent density-functional theory for the calculation of excitation energies of large molecules” *J. Chem. Phys.* **1998**, *109*, 8218–8224.

[96] C. Van Caillie, R. D. Amos, “Geometric derivatives of excitation energies using SCF and DFT” *Chem. Phys. Lett.* **1999**, *308*, 249–255.

[97] C. Van Caillie, R. D. Amos, “Geometric derivatives of density functional theory excitation energies using gradient-corrected functionals” *Chem. Phys. Lett.* **2000**, *317*, 159–164.

[98] F. Furche, R. Ahlrichs, “Adiabatic time-dependent density functional methods for excited state properties” *J. Chem. Phys.* **2002**, *117*, 7433–7447.

[99] G. Scalmani, M. J. Frisch, B. Mennucci, J. Tomasi, R. Cammi, V. Barone, “Geometries and properties of excited states in the gas phase and in solution: Theory and application of a time-dependent density functional theory polarizable continuum model” *J. Chem. Phys.* **2006**, *124*, 094–107.

[100] N. M. O'Boyle, A. L. Tenderholt, K. M. Langner, “cclib: A library for package-independent computational chemistry algorithms” *J. Comput. Chem.* **2008**, *29*, 839–845.

[101] G. Knizia, “Intrinsic Atomic Orbitals: An Unbiased Bridge between Quantum Theory and Chemical Concepts” *J. Chem. Theory Comput.* **2013**, *9*, 4834–4843.

[102] F. Neese, “The ORCA Program System” *WIREs Comput. Mol. Sci.* **2012**, 2, 73–78.
